# Stability Analysis on Magnetohydrodynamic Flow of Casson Fluid over a Shrinking Sheet with Homogeneous-Heterogeneous Reactions

**DOI:** 10.3390/e20090652

**Published:** 2018-08-30

**Authors:** Rusya Iryanti Yahaya, Norihan Md Arifin, Siti Suzilliana Putri Mohamed Isa

**Affiliations:** 1 Institute for Mathematical Research, Universiti Putra Malaysia, Serdang Selangor 43400 UPM, Malaysia; 2 Department of Mathematics, Universiti Putra Malaysia, Serdang Selangor 43400 UPM, Malaysia; 3 Centre of Foundation Studies For Agricultural Science, Universiti Putra Malaysia, Serdang Selangor 43400 UPM, Malaysia

**Keywords:** stability analysis, homogeneous-heterogeneous, MHD, shrinking sheet, Casson fluid

## Abstract

Two-dimensional magnetohydrodynamic (MHD) stagnation point flow of incompressible Casson fluid over a shrinking sheet is studied. In the present study, homogeneous-heterogeneous reactions, suction and slip effects are considered. Similarity variables are introduced to transform the governing partial differential equations into non-linear ordinary differential equations. The transformed equations and boundary conditions are then solved using the bvp4c solver in MATLAB. The local skin friction coefficient is tabulated for different values of suction and shrinking parameters. The profiles for fluid velocity and concentration for various parameters are illustrated. It was found that two solutions were obtained at certain ranges of parameters. Then, the bvp4c solver was used to perform stability analysis on the dual solutions. Based on the results, the first solution was more stable and physically meaningful than the other solution. The skin friction coefficient increased when suction increased, but decreased when the magnitude of shrinking parameter increased. Meanwhile, the velocity and concentration profile increased in the presence of a magnetic field. It is also noted that the higher the strength of the homogeneous-heterogeneous reactions, the lower the concentration of reactants.

## 1. Introduction

Casson fluid is a shear-thinning non-Newtonian fluid that exhibits yield stress, the stress that must be exceeded to make the fluid flow. The fluid acts like a solid and does not flow unless the applied shear stress is larger than the yield stress. Tomato sauce, jelly, honey and even human blood are some of the examples. Many studies have been made on Casson fluid. One of the studies was by Mukhopadhay [1] on the boundary layer flow over a nonlinear stretching surface. Then, this study was extended by Pramanik [2] by considering thermal radiation and an exponentially-stretching surface for the boundary. Recently, the unsteady case was discussed by Khan et al. [3].

The study on the motion of electrically-conducting fluid in the presence of a magnetic field is called magnetohydrodynamics (MHD). In engineering, the application of MHD can be seen in the electromagnetic pump. The pumping motion of this device is caused by the Lorentz force. This force is produced when mutually perpendicular magnetic fields and electric currents are applied in the direction perpendicular to the axis of a pipe containing conducting fluid. Besides that, MHD principles are also used in designing heat exchangers, generators and accelerators. It was found that the strength of magnetic field can affect the fluid flow and temperature distributions inside an enclosure [4]. The study of entropy generation in MHD flow of nanofluid over a rotating disk was done by Rashidi et al. [5]. This study helped to determine the feasibility of using magnetic rotating disk drives in nuclear space propulsion engines. The nanofluid was found to be useful in enhancing the heat transfer rate. Further discussions on the rheological behavior and heat transfer enhancement of nanofluids can be found in the studies by Karimipour et al. [6], Esfe et al. [7], Karimipour et al. [8], Bahrami et al. [9] and Zadkhast et al. [10]. Then, Rashidi et al. [11] extended the study for fluid with variable properties and slip effects. There were also studies on MHD flow of Casson fluid. Nadeem et al. [12] studied the MHD flow with an exponentially-shrinking sheet as the boundary. Raju et al. [13] then discussed the flow over an exponentially-stretching sheet. Thermal radiation, viscous dissipation and chemical reaction were considered in the study. Later, Reddy [14] extended this study by excluding the viscous dissipation effect, and the surface was considered as an inclined stretching surface. The MHD stagnation point flow was discussed by Medikare et al. [15] where the viscous dissipation effect was taken into account in the study. On the other hand, Qing et al. [16] did a study on MHD flow of Casson nanofluid by considering thermal radiation and chemical reactions.

Homogeneous and heterogeneous reactions take place in many chemical reacting systems. Homogeneous reaction occurs at the same phase in bulk of the fluid, whereas heterogeneous reaction occurs at two or more phases commonly on the catalytic surfaces. One of the earliest study was done by Merkin [17]. He studied the isothermal homogeneous-heterogeneous reactions in boundary layer flow over a flat surface. It was found that homogeneous reaction dominates downstream. This leads to other studies related to homogeneous-heterogeneous reactions including the study by Rana et al. [18] on the mixed convection oblique flow of Casson fluid in the presence of internal heating. Then, the study of the reactions on stagnation point flow was done by Raju et al. [19] where the induced magnetic field effect was considered. It was found that the induced magnetic field helped to improve the heat transfer rate. Sheikh and Abbas [20] discussed the reactions on flow over a stretching and shrinking surfaces in the presence of uniform suction and slip effects. Next, Khan et al. [21] expanded the study by Raju et al. [19] into MHD flow with the homogeneous heat effect and omitting the induced magnetic field. The magnetic parameter was found to reduce the velocity. Recently, Khan et al. [22] did a study on a flow with viscous dissipation and Joule heating.

Nowadays, there has been growing interest in the stability analysis of dual solutions. The analysis helps determine the stability and significance of the solutions to the problem. Merkin [23] performed stability analysis on both of the solutions obtained in the study of mixed convection. By considering the unsteady problem, it was found that the upper branch of the solutions was stable while the lower branch was not stable. The same conclusion was obtained in the study by Weidman et al. [24]. Mahapatra and Nandy [25] performed stability analysis on the solutions obtained in stagnation point flow of viscous fluid over a nonlinear shrinking surface. Then, in the study by Roşca and Pop [26], the stability analysis was performed using the bvp4c solver in MATLAB. Next, the analysis was also done by Awaludin et al. [27] on the dual solutions obtained in the shrinking sheet case of stagnation point flow.

Based on the previous research, this study will extend the problem proposed by Sheikh and Abbas [20] to the case of magnetohydrodynamic flow over a shrinking sheet. The differential equations and the boundary conditions will be solved using the bvp4c solver in MATLAB. Stability analysis will then be performed on the dual solutions obtained in this study.

## 2. Materials and Methods

### 2.1. Mathematical Formulation

**Figure 1:**
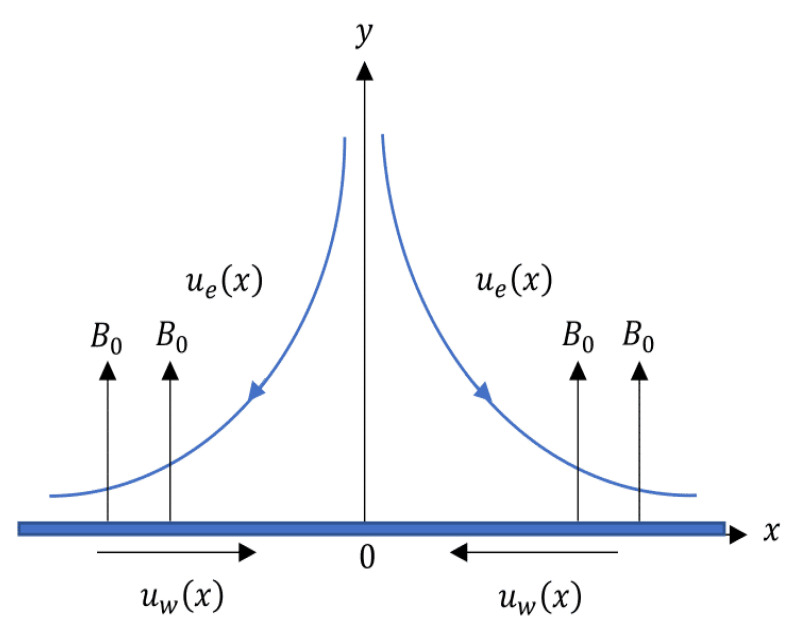
Geometry of the problem.

Consider a steady flow of Casson fluid towards a shrinking sheet. Slip effects are considered at the boundary. The Cartesian plane is used to represent the problem such that the shrinking sheet is placed along the *x*-axis, as shown in Figure 1. The fluid flow is assumed to be confined in the region y>0 towards the sheet with x=0 as the stagnation point. The sheet is shrunk linearly with a velocity of $u_{w}\left(x\right)=mx$ where m<0 is for the shrinking case and the velocity of external flow is $u_{e}\left(x\right)=cx$ where c>0 is the strength of stagnation flow. A magnetic field with strength $B_{0}$ is applied in the direction perpendicular to the sheet (*y*-direction). The induced magnetic field is neglected in this study due to the assumption of a small magnetic Reynolds number.

In the present study, homogeneous-heterogeneous reactions involving two chemical species *A* and *B* in the boundary layer flow of Casson fluid near a stagnation point are considered. The problem is analyzed based on a simple model proposed by Merkin [17]. The homogeneous and heterogeneous reactions can be represented by the following equations, respectively,

(1) $$\begin{array}{cc} A+2B & \to 3B,\;\;\;\;\;\;rate=k_{c}ab^{2}, \end{array}$$

(2) $$\begin{array}{cc} A & \to B,\;\;\;\;\;\;\;\;\;rate=k_{s}a, \end{array}$$

where $k_{c}$ and $k_{s}$ are the rate constants, while *a* and *b* are the respected concentrations of chemical species *A* and *B*. The two reaction processes are assumed to be isothermal with a concentration, $a_{0}$ for reactant *A* and no autocatalyst *B* in the ambient fluid.

Based on the stated assumptions, the governing equations are:

(3) $$\begin{array}{cc} \frac{\partial u}{\partial x}+\frac{\partial v}{\partial y} & =0, \end{array}$$

(4) $$\begin{array}{cc} u\frac{\partial u}{\partial x}+v\frac{\partial u}{\partial y} & =u_{e}\frac{du_{e}}{dx}+\nu \left(1+\frac{1}{\beta }\right)\frac{\partial^{2}u}{\partial y^{2}}+\frac{\sigma B_{0}^{2}}{\rho }\left(u_{e}-u\right), \end{array}$$

(5) $$\begin{array}{cc} u\frac{\partial a}{\partial x}+v\frac{\partial a}{\partial y} & =D_{A}\frac{\partial^{2}a}{\partial y^{2}}-k_{c}ab^{2}, \end{array}$$

(6) $$\begin{array}{cc} u\frac{\partial b}{\partial x}+v\frac{\partial b}{\partial y} & =D_{B}\frac{\partial^{2}b}{\partial y^{2}}+k_{c}ab^{2}, \end{array}$$

with the boundary conditions:

(7) $$\begin{array}{c} u\left(0\right)=u_{w}\left(x\right)=mx+L\left(1+\frac{1}{\beta }\right)\frac{\partial u}{\partial y},\;\;\;\;\;v\left(0\right)=-v_{w},\\ D_{A}\frac{\partial a}{\partial y}{|}_{y=0}=k_{s}a\left(0\right),\;\;\;\;\;D_{B}\frac{\partial b}{\partial y}{|}_{y=0}=-k_{s}a\left(0\right),\\ u\left(\infty \right)=u_{e}\left(x\right)=cx,\;\;\;\;\;a\left(\infty \right)=a_{0},\;\;\;\;\;b\left(\infty \right)=0, \end{array}$$

where *u* and *v* are the velocity components in the *x*- and *y*-directions, respectively, ν is the kinematic viscosity, σ is the electrical conductivity of the fluid, ρ is the density, $D_{A}$ and $D_{B}$ are the respective diffusion coefficients for species *A* and *B*, *m* and *c* are constants with dimension (time)${}^{-1}$, *L* is the velocity slip length parameter, $v_{w}$ is the constant mass transfer velocity and $\beta =\mu_{B}\sqrt{2\pi_{c}}/p_{y}$ is the Casson parameter where $\mu_{B}$ is the plastic dynamic viscosity of Casson fluid, $p_{y}$ is the yield stress and $\pi_{c}$ is the critical value for the product of the component of the deformation rate with itself.

The following dimensionless variables are introduced:

(8) $$\begin{array}{c} \eta =\sqrt{\frac{c}{\nu }}y,\;\;\;\;\;u=cxf^{\prime }\left(\eta \right),\;\;\;\;\;v=-\sqrt{c\nu }f\left(\eta \right),\;\;\;\;\;g\left(\eta \right)=\frac{a}{a_{0}},\;\;\;\;\;h\left(\eta \right)=\frac{b}{a_{0}}, \end{array}$$

for similarity transformations of the governing equations. Substituting (8) into Equations (3)–(7) will result in the the following ordinary differential equations and boundary conditions:

(9) $$\begin{array}{c} \left(1+\frac{1}{\beta }\right)f^{\prime \prime \prime }+ff^{\prime \prime }-f^{\prime 2}+M^{2}\left(1-f^{\prime }\right)+1=0, \end{array}$$

(10) $$\begin{array}{c} \frac{1}{Sc}g^{\prime \prime }+fg^{\prime }-Kgh^{2}=0, \end{array}$$

(11) $$\begin{array}{c} \frac{\delta }{Sc}h^{\prime \prime }+fh^{\prime }+Kgh^{2}=0, \end{array}$$

(12) $$\begin{array}{c} f\left(0\right)=S,\;\;\;\;\;f^{\prime }\left(0\right)=\lambda +\upsilon \left(1+\frac{1}{\beta }\right)f^{\prime \prime }\left(0\right),\;\;\;\;\;f^{\prime }\left(\infty \right)=1, \end{array}$$

(13) $$\begin{array}{c} g^{\prime }\left(0\right)=K_{s}g\left(0\right),\;\;\;\;\;g\left(\infty \right)=1, \end{array}$$

(14) $$\begin{array}{c} \delta h^{\prime }\left(0\right)=-K_{s}g\left(0\right),\;\;\;\;\;h\left(\infty \right)=0. \end{array}$$

In the above equations, the prime indicates the ordinary derivative with respect to η. $M^{2}=\frac{\sigma B_{0}^{2}}{\rho c}$ is the magnetic parameter; $Sc=\frac{\nu }{D_{A}}$ is the Schmidt number; $K=\frac{k_{c}a_{0}^{2}}{c}$ is the strength of homogeneous reaction; while $K_{s}=\frac{k_{s}Re^{-1/2}}{D_{A}}$ is the strength of heterogeneous reaction with the Reynolds number, $Re=\frac{c}{\nu }$; $\delta =\frac{D_{B}}{D_{A}}$ is the ratio of diffusion coefficients; $S=\frac{v_{w}}{\sqrt{c\nu }}$ is the mass suction parameter with S>0; $\lambda =\frac{m}{c}$ is the ratio of the shrinking rate to the rate of external flow with λ<0 for a shrinking sheet; and $\upsilon =L\sqrt{\frac{c}{\nu }}$ is the velocity slip parameter.

Chaudhary and Merkin [28] stated that the diffusion coefficients of species *A* and *B* are of a comparable size in most applications. Thus, it can be assumed that $D_{A}$ is equal to $D_{B}$ and δ=1. Therefore, Equations (10) and (11) can be reduced to:

(15) $$\begin{array}{c} \frac{1}{Sc}g^{\prime \prime }+fg^{\prime }-Kg{\left(1-g\right)}^{2}=0, \end{array}$$

and the boundary conditions are reduced to:

(16) $$\begin{array}{c} g^{\prime }\left(0\right)=K_{s}g\left(0\right),\;\;\;\;\;g\left(\infty \right)=1. \end{array}$$

The transformed equations and boundary conditions are then solved using the bvp4c solver in MATLAB. A detailed derivation of Equations (9)–(16) is provided in Appendix A.

The dimensionless skin friction coefficient $C_{f}$ is given by:

$$\begin{array}{c} Re_{x}^{1/2}C_{f}=\left(1+\frac{1}{\beta }\right)f^{\prime \prime }\left(0\right), \end{array}$$

with $Re_{x}=xu_{e}/\nu$ as the local Reynolds number.

### 2.2. Stability Analysis

Stability analysis is performed by considering the problem as an unsteady problem. Equations (4)–(6) are replaced by:

(17) $$\begin{array}{cc} \frac{\partial u}{\partial t}+u\frac{\partial u}{\partial x}+v\frac{\partial u}{\partial y} & =u_{e}\frac{\partial u_{e}}{\partial x}+\nu \left(1+\frac{1}{\beta }\right)\frac{\partial^{2}u}{\partial y^{2}}+\frac{\sigma B_{0}^{2}}{\rho }\left(u_{e}-u\right), \end{array}$$

(18) $$\begin{array}{cc} \frac{\partial a}{\partial t}+u\frac{\partial a}{\partial x}+v\frac{\partial a}{\partial y} & =D_{A}\frac{\partial^{2}a}{\partial y^{2}}-k_{c}ab^{2}, \end{array}$$

(19) $$\begin{array}{cc} \frac{\partial b}{\partial t}+u\frac{\partial b}{\partial x}+v\frac{\partial b}{\partial y} & =D_{B}\frac{\partial^{2}b}{\partial y^{2}}+k_{c}ab^{2}, \end{array}$$

in which *t* represents time. New dimensionless variables are introduced to replace the variables in (8),

(20) $$\begin{array}{c} \begin{array}{cc} \eta =\sqrt{\frac{c}{\nu }}y,\;\;\;\;\;u=cxf^{\prime }\left(\eta ,\tau \right),\;\;\;\;\;v & =-\sqrt{c\nu }f\left(\eta ,\tau \right),\;\;\;\;\;g\left(\eta ,\tau \right)=\frac{a}{a_{0}},\\ h(\eta ,\tau ) & =\frac{b}{a_{0}},\;\;\;\;\;\;\tau =ct, \end{array} \end{array}$$

so that Equations (9)–(11) can be replaced by:

(21) $$\begin{array}{cc} \left(1+\frac{1}{\beta }\right)\frac{\partial^{3}f}{\partial \eta^{3}}+f\frac{\partial^{2}f}{\partial \eta^{2}}-{\left(\frac{\partial f}{\partial \eta }\right)}^{2}+M^{2}\left(1-\frac{\partial f}{\partial \eta }\right)+1-\frac{\partial^{2}f}{\partial \eta \partial \tau } & =0, \end{array}$$

(22) $$\begin{array}{cc} \frac{1}{Sc}\frac{\partial^{2}g}{\partial \eta^{2}}+f\frac{\partial g}{\partial \eta }-Kgh^{2}-\frac{\partial g}{\partial \tau } & =0, \end{array}$$

(23) $$\begin{array}{cc} \frac{\delta }{Sc}\frac{\partial^{2}h}{\partial \eta^{2}}+f\frac{\partial h}{\partial \eta }+Kgh^{2}-\frac{\partial h}{\partial \tau } & =0, \end{array}$$

and the boundary conditions become:

(24) $$\begin{array}{cc} \frac{\partial f}{\partial \eta }\left(0,\tau \right) & =\lambda +\upsilon \left(1+\frac{1}{\beta }\right)\frac{\partial^{2}f}{\partial \eta^{2}}\left(0,\tau \right),\;\;\;\;\;f\left(0,\tau \right)=S,\;\;\;\;\;\frac{\partial f}{\partial \eta }\left(\infty ,\tau \right)=1, \end{array}$$

(25) $$\begin{array}{cc} \frac{\partial g}{\partial \eta }\left(0,\tau \right) & =K_{s}g\left(0,\tau \right),\;\;\;\;\;\;\;\;\;g\left(\infty ,\tau \right)=1, \end{array}$$

(26) $$\begin{array}{cc} \delta \frac{\partial h}{\partial \eta }\left(0,\tau \right) & =-K_{s}g\left(0,\tau \right),\;\;\;\;h\left(\infty ,\tau \right)=0. \end{array}$$

Then, Equations (22) and (23) are reduced to the following equation:

(27) $$\begin{array}{c} \frac{1}{Sc}\frac{\partial^{2}g}{\partial \eta^{2}}+f\frac{\partial g}{\partial \eta }-Kg{\left(1-g\right)}^{2}-\frac{\partial g}{\partial \tau }=0, \end{array}$$

using the previous assumption where $D_{A}$ is equal to $D_{B}$ and δ=1. The boundary conditions are given by:

(28) $$\begin{array}{c} \frac{\partial g}{\partial \eta }\left(0,\tau \right)=K_{s}g\left(0,\tau \right),\;\;\;\;\;g\left(\infty ,\tau \right)=1. \end{array}$$

According to Harris et al. [29], in order to test the stability of the steady flow solution $f\left(\eta \right)=f_{0}\left(\eta \right)$ and $g\left(\eta \right)=g_{0}\left(\eta \right)$ that satisfies the boundary value problem (9), (12), (15) and (16), we write:

(29) $$\begin{array}{c} f\left(\eta ,\tau \right)=f_{0}\left(\eta \right)+e^{-\gamma \tau }F\left(\eta ,\tau \right),\\ g\left(\eta ,\tau \right)=g_{0}\left(\eta \right)+e^{-\gamma \tau }G\left(\eta ,\tau \right), \end{array}$$

where F(η,τ) and G(η,τ) are small relative to $f_{0}\left(\eta \right)$ and $g_{0}\left(\eta \right)$ and γ is the unknown eigenvalue parameter. Awaludin et al. [27] stated that Equations (21) and (27) along boundary conditions (24) and (28) will yield infinite sets of eigenvalues $\gamma_{1}<\gamma_{2}<\gamma_{3}<\gamma_{4}<\ldots$. If the smallest eigenvalue γ<0, there is initial growth of disturbance in the flow and the flow becomes unstable, whereas if γ>0, there is initial decay that does not interrupt the flow and the flow is said to be stable. Substituting (29) into Equations (21) and (27) yields the following linearized equations:

(30) $$\begin{array}{cc} \left(1+\frac{1}{\beta }\right)\frac{\partial^{3}F}{\partial \eta^{3}}+f_{0}\frac{\partial^{2}F}{\partial \eta^{2}}+F\frac{\partial^{2}f_{0}}{\partial \eta^{2}}-\left(2\frac{\partial f_{0}}{\partial \eta }+M^{2}-\gamma \right)\frac{\partial F}{\partial \eta }-\frac{\partial^{2}F}{\partial \eta \partial \tau } & =0, \end{array}$$

(31) $$\begin{array}{c} \frac{1}{Sc}\frac{\partial^{2}G}{\partial \eta^{2}}+F\frac{\partial g_{0}}{\partial \eta }+f_{0}\frac{\partial G}{\partial \eta }-\left(K-4Kg_{0}+3Kg_{0}^{2}-\gamma \right)G-\frac{\partial G}{\partial \tau }=0, \end{array}$$

and the boundary conditions:

(32) $$\begin{array}{c} F\left(0,\tau \right)=0,\;\;\;\;\;\frac{\partial F}{\partial \eta }\left(0,\tau \right)=\upsilon \left(1+\frac{1}{\beta }\right)\frac{\partial^{2}F}{\partial \eta^{2}}\left(0,\tau \right),\;\;\;\;\;\frac{\partial G}{\partial \eta }\left(0,\tau \right)=K_{s}G\left(0,\tau \right), \end{array}$$

(33) $$\begin{array}{c} \frac{\partial F}{\partial \eta }\left(\eta ,\tau \right)\to 0,\;\;\;\;\;G\left(\eta ,\tau \right)\to 0\;\;\;\;\mathrm{as}\;\;\;\;\;\eta \to \infty . \end{array}$$

Based on Weidman et al. [24], the initial growth or decay in (29) can be identified by setting τ to 0 so that $F=F_{0}\left(\eta \right)$ and $G=G_{0}\left(\eta \right)$ in Equations (30)–(33). Then, the following linear eigenvalue problem is obtained:

(34) $$\begin{array}{cc} \left(1+\frac{1}{\beta }\right)F_{0}^{\prime \prime \prime }+f_{0}F_{0}^{\prime \prime }+f_{0}^{\prime \prime }F_{0}-\left(2f_{0}^{\prime }+M^{2}-\gamma \right)F_{0}^{\prime } & =0, \end{array}$$

(35) $$\begin{array}{cc} \frac{1}{Sc}G_{0}^{\prime \prime }+g_{0}^{\prime }F_{0}+f_{0}G_{0}^{\prime }-\left(K-4Kg_{0}+3Kg_{0}^{2}-\gamma \right)G_{0} & =0, \end{array}$$

together with the boundary conditions:

(36) $$\begin{array}{c} F_{0}\left(0\right)=0,\;\;\;\;\;\;F_{0}^{\prime }\left(0\right)=\upsilon \left(1+\frac{1}{\beta }\right)F_{0}^{\prime \prime }\left(0\right),\;\;\;\;\;\;G_{0}^{\prime }\left(0\right)=K_{s}G_{0}\left(0\right), \end{array}$$

(37) $$\begin{array}{c} F_{0}^{\prime }\left(\eta \right)\to 0,\;\;\;\;\;\;G_{0}\left(\eta \right)\to 0\;\;\;\;\mathrm{as}\;\;\;\;\;\eta \to \infty . \end{array}$$

Then, $F_{0}^{\prime }\left(\eta \right)\to 0$ as η→∞ are chosen to relax. This is because Harris et al. [29] stated that the range of possible eigenvalues can be obtained by relaxing one of the boundary conditions, $F_{0}\left(\eta \right)$ or $G_{0}\left(\eta \right)$. Thus, Equations (34) and (35) are solved along the new boundary conditions,

(38) $$\begin{array}{c} F_{0}\left(0\right)=0,\;\;\;\;\;F_{0}^{\prime }\left(0\right)=\upsilon \left(1+\frac{1}{\beta }\right)F_{0}^{\prime \prime }\left(0\right),\;\;\;\;\;G_{0}^{\prime }\left(0\right)=K_{s}G_{0}\left(0\right),\;\;\;\;\;F_{0}^{\prime \prime }\left(0\right)=1, \end{array}$$

(39) $$\begin{array}{c} G_{0}\left(\eta \right)\to 0\;\;\;\;\;\mathrm{as}\;\;\;\;\;\eta \to \infty . \end{array}$$

The eigenvalues are computed using the bvp4c solver in MATLAB. The smallest eigenvalue is the eigenvalue with the smallest error. A detailed derivation of Equations (21)–(39) is presented in Appendix B.

### 2.3. Numerical Method

The nonlinear ordinary differential equations, Equations (9) and (15) subject to the boundary conditions (12) and (16) are solved by using the bvp4c solver in MATLAB. This solver has been widely used by other researchers to solve the boundary value problem. The solver is a finite difference code with fourth order accuracy. In order to use the solver, the equations have to be rewritten as a set of equivalent first order ordinary differential equations. This is done by using the following substitutions:

For Equation (9),

(40) $$\begin{array}{cc} y(1) & =f,\\ y{\left(1\right)}^{\prime } & =f^{\prime }=y\left(2\right),\\ y{\left(2\right)}^{\prime } & =f^{\prime \prime }=y\left(3\right),\\ y{\left(3\right)}^{\prime } & =f^{\prime \prime \prime }=\frac{\left[-y\left(1\right)y\left(3\right)+{\left(y\left(2\right)\right)}^{2}-M^{2}\left(1-y\left(2\right)\right)-1\right]}{\left[1+\frac{1}{\beta }\right]}. \end{array}$$

For Equation (15),

(41) $$\begin{array}{cc} y(4) & =g,\\ y{\left(4\right)}^{\prime } & =g^{\prime }=y\left(5\right),\\ y{\left(5\right)}^{\prime } & =g^{\prime \prime }=Sc\left[-y\left(1\right)y\left(5\right)+Ky\left(4\right){\left(1-y\left(4\right)\right)}^{2}\right]. \end{array}$$

For the boundary conditions (12) and (16),

(42) $$\begin{array}{cc} ya(1) & =S,\;\;\;\;\;ya\left(2\right)=\lambda +\upsilon \left(1+\frac{1}{\beta }\right)ya\left(3\right),\;\;\;\;\;yb\left(2\right)=1, \end{array}$$

(43) $$\begin{array}{cc} ya(5) & =K_{s}ya\left(4\right),\;\;\;\;\;yb\left(4\right)=1. \end{array}$$

Equations (40)–(43) are then coded into the bvp4c solver. The syntax of the solver, sol = bvp4c(@OdeBVP,@OdeBc,solinit,options) contains the function handle @OdeBVP, into which the Equations (40) and (41) are coded. Then, the boundary conditions (42) and (43) are coded into the function handle @OdeBC.

The bvp4c solver applies the collocation formula of three-stage Lobatto IIIa formula. Bilal et al. [30] states that the collocation polynomials provide a $C^{1}$-continuous solution with fourth order accuracy uniformly in the interval of integration. The interval of integration is set according to the boundary conditions. In this technique, the interval of integration is divided into the subinterval by using a mesh of points. Then, a system of algebraic equations, resulting from the boundary conditions and the collocation conditions imposed on all the subintervals, are solved to obtain the numerical solution. The solver estimates the error of the numerical solution on each of the subintervals. The process is repeated if the solution fails to satisfy the tolerance criteria. The points of the initial mesh and initial approximation of the solution at the mesh points are coded in solinit, while the options is an optional integration argument. The solver is ran, and the results are printed out in the form of numerical solutions and graphs. When the other guess value in the structure solinit results in other solutions that also satisfy the boundary conditions, this indicates that multiple solutions exist to the problem.

The same procedures are done for stability analysis. New substitutions are introduced to rewrite Equations (34) and (35) and the boundary conditions (38) and (39) into first order ordinary differential equations. Let,

$$\begin{array}{ccccc} y(1) & =F_{0}, & \mathrm{and} & s(1) & =f_{0},\\ y(2) & =F_{0}^{\prime }, & & s(2) & =f_{0}^{\prime },\\ y(3) & =F_{0}^{\prime \prime }, & & s(3) & =f_{0}^{\prime \prime },\\ y(4) & =G_{0}, & & s(4) & =g_{0},\\ y(5) & =G_{0}^{\prime }, & & s(5) & =g_{0}^{\prime }. \end{array}$$

The new first order ordinary differential equations are then coded into the solver. The smallest eigenvalue, γ, is computed by setting an appropriate step size at a command in the solver.

## 3. Results and Discussion

In the present study, the results on the effects of suction parameter *S*, shrinking parameter λ, Casson parameter β, slip parameter υ, magnetic parameter *M*, the strength of homogeneous reaction parameter *K*, the strength of heterogeneous reaction parameter $K_{s}$ and Schmidt number Sc on the fluid velocity and concentration are illustrated in graphs. Besides that, the variation of local skin friction coefficient $f^{\prime \prime }\left(0\right)$ on various values of *S* and the effect of *S* on the concentration gradient $g^{\prime }\left(0\right)$ are also shown in the form of graphs. All the results obtained in this study were of two solutions or also known as the dual solutions. These solutions were obtained at certain ranges of parameters.

Equations (9) and (15) together with the boundary conditions (12) and (16) were solved numerically using the bvp4c solver in MATLAB. The validity of the method used was then checked by comparing the obtained results of $f^{\prime \prime }\left(0\right)$ with the results from previous studies, as shown in Table 1. The computations of $f^{\prime \prime }\left(0\right)$ in Kameswaran et al. [31] and Bhattacharyya [32] were done by using the bvp4c solver and shooting method, respectively. Thus, the usage of these studies in validating the method used in the present study was suitable. It was found that the results were in good agreement. This reassured that the method used was accurate. In this paper, the computations were done for various values of parameters involved in the flow equations. All the results obtained were in the form of dual solutions. The velocity profiles, concentration profiles, the graph of skin friction and concentration gradient at the surface were plotted.

**Figure 2:**
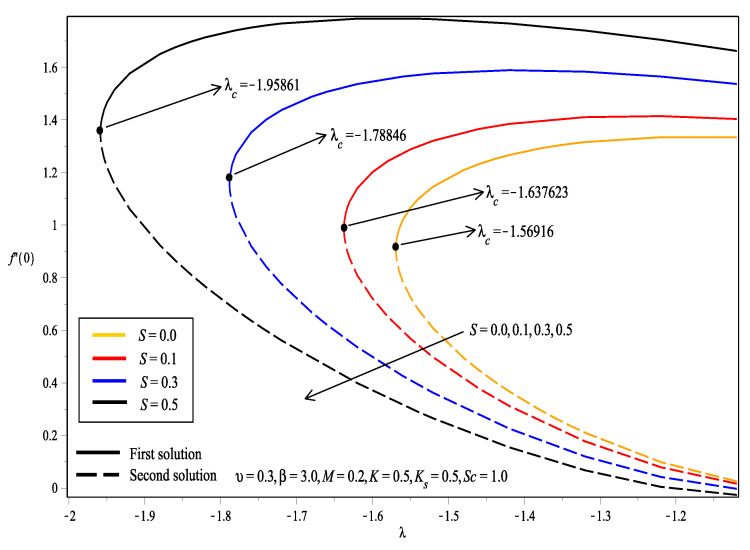
Variation of skin friction coefficient, $f^{\prime \prime }\left(0\right)$, for different values of suction parameter, *S*.

Figure 2 shows that dual solutions existed when $\lambda >\lambda_{c}$, while no solution was obtained when $\lambda <\lambda_{c}$. $\lambda_{c}$ is the critical point where the solution at this point is unique. The value of $f^{\prime \prime }\left(0\right)$ for the first solution was observed to increase as *S* increased, which agrees with the results obtained in Table 2. This shows that the increase in suction caused the wall shear stress to increase. Likewise, the value of $|\lambda_{c}|$ increased as *S* increased. It is also noted that the value of $f^{\prime \prime }\left(0\right)$ was always positive. This denotes that the fluid exerted drag force on the sheet.

**Figure 3:**
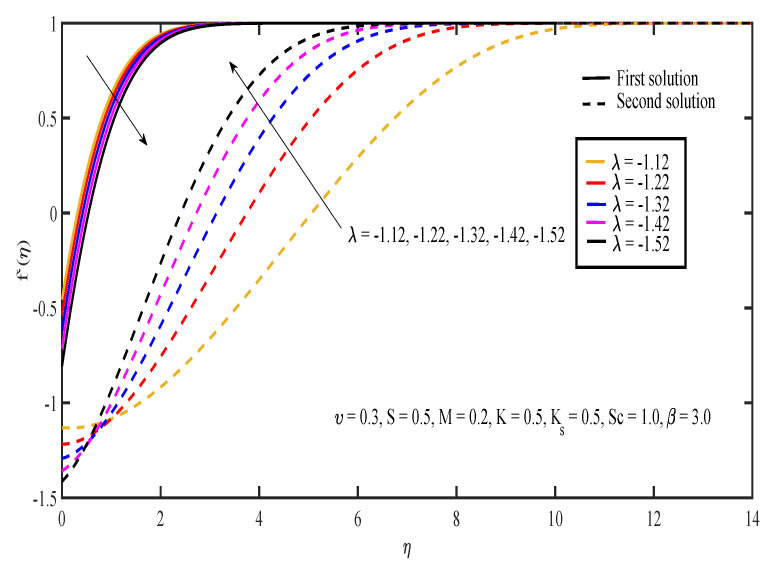
Velocity profiles for different values of shrinking parameter, λ.

**Figure 4:**
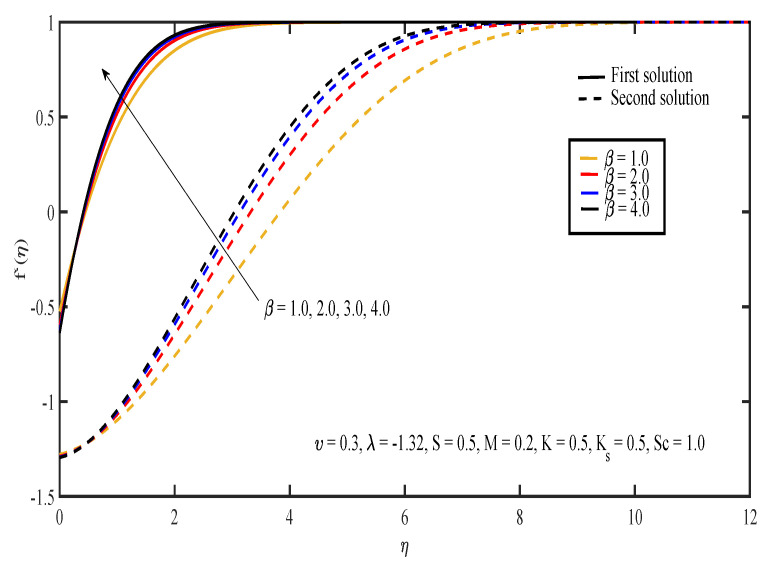
Velocity profiles for different values of Casson parameter, β.

**Figure 5:**
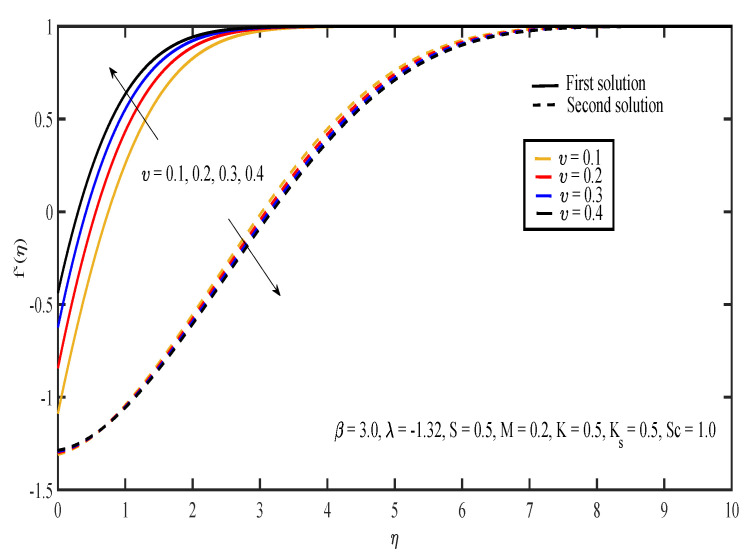
Velocity profiles for different values of slip parameter, υ.

**Figure 6:**
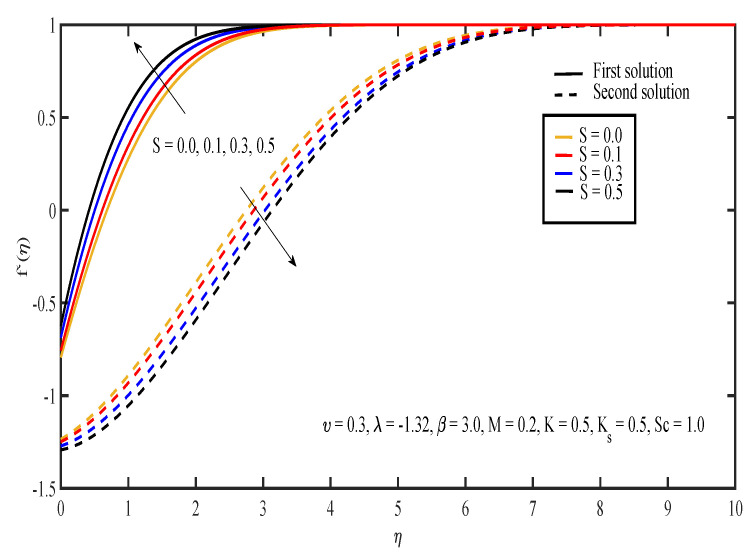
Velocity profiles for different values of suction parameter, *S*.

**Figure 7:**
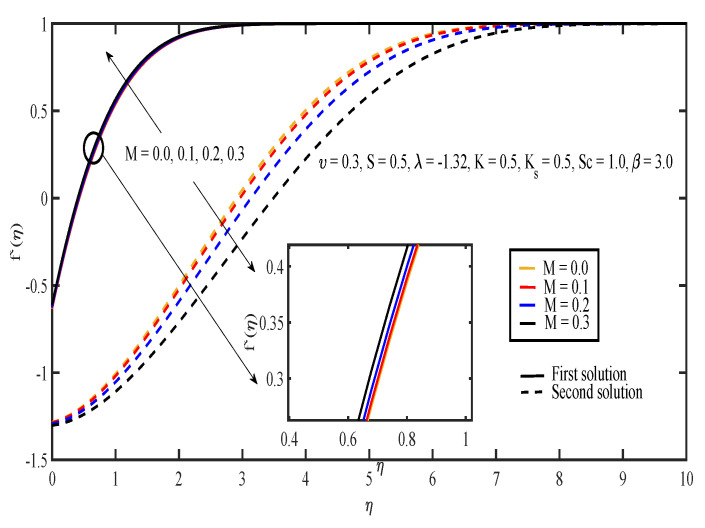
Velocity profiles for different values of magnetic parameter, *M*.

Figure 3, Figure 4, Figure 5, Figure 6 and Figure 7 display the effects of different parameters on the dimensionless fluid velocity, $f^{\prime }\left(\eta \right)$. The values of $f^{\prime }\left(\eta \right)$ are noted to be initially negative. Then, $f^{\prime }\left(\eta \right)$ became positive as η increased. The effect of λ on $f^{\prime }\left(\eta \right)$ is shown in Figure 3. The increasing value of |λ| caused the value of $f^{\prime }\left(\eta \right)$ to decrease in the first solution and increase in the other solution. The thickness of the momentum boundary layer for the first solution is noted to become larger as |λ| increases. In Figure 4, the increase in β caused $f^{\prime }\left(\eta \right)$ to increase and the thickness of momentum boundary layer to decrease in both of the solutions. The large value of β indicated that the yield stress was smaller compared to the viscosity and deformation rate. Sheikh and Abbas [20] stated that the yield stress became less when the value of the Casson parameter increased and caused the thickness of the momentum boundary layer to decrease. The presence of tensile stress due to elasticity led to the contraction of boundary layer thickness. Next, Figure 5 shows that the increase in υ caused $f^{\prime }\left(\eta \right)$ of the first solution to increase. The opposite results were obtained for the other solution except for a small part near the surface of the sheet. On the other hand, the effect of *S* on $f^{\prime }\left(\eta \right)$ is shown in Figure 6. Suction was one of the methods used in delaying the flow separation. Miklavčič and Wang [33] stated that enough suction was needed to maintain the fluid flow on the shrinking surfaces. The presence of suction caused the fluid velocity in the first solution to increase, while the velocity in the other solution reduced. In the first solution, the thickness of the boundary layer reduced as suction increased. The variation of velocity profile with *M* is illustrated in Figure 7. It can be noted that the higher the value of *M*, the higher the fluid velocity in the first solution. However, different results were obtained for the second solution where the increase in the value of *M* caused the velocity to decrease. In the presence of magnetic field, a drag-like force called the Lorentz force is produced. This force provides resistance to the momentum of the fluid particles. This will make the boundary layer thickness to become thinner. In Figure 7, it can be noticed that the thickness of the boundary layer for the first solution becomes thinner as *M* increased. The boundary layer thickness of the second solution was significantly bigger than the first solution.

**Figure 8:**
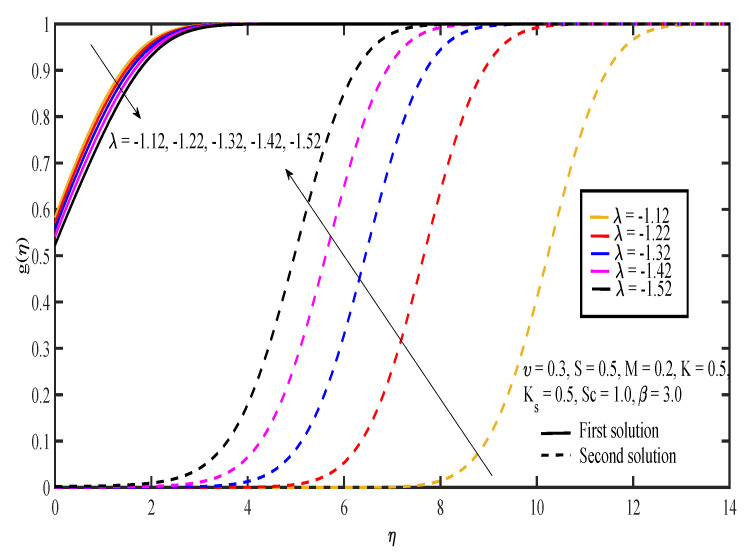
Concentration profiles for different values of shrinking parameter, λ.

**Figure 9:**
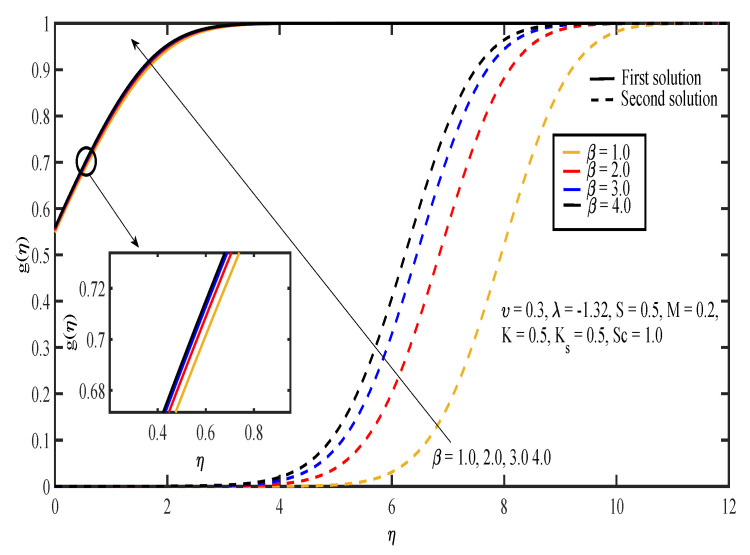
Concentration profiles for different values of Casson parameter, β.

**Figure 10:**
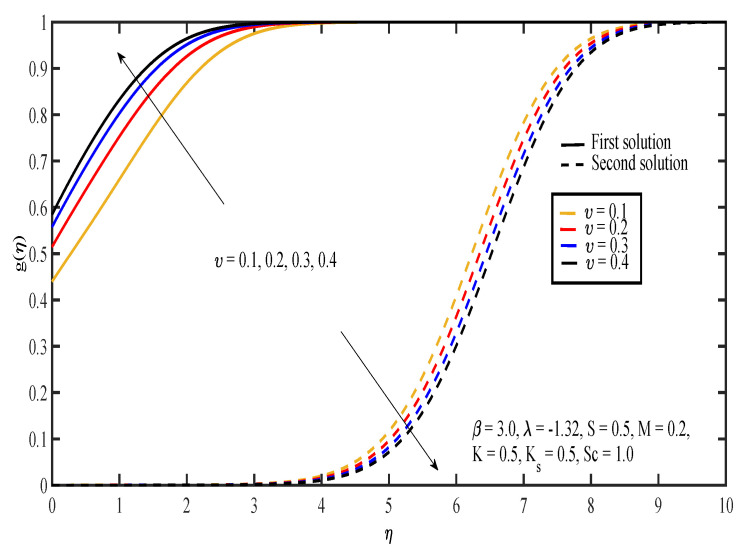
Concentration profiles for different values of slip parameter, υ.

**Figure 11:**
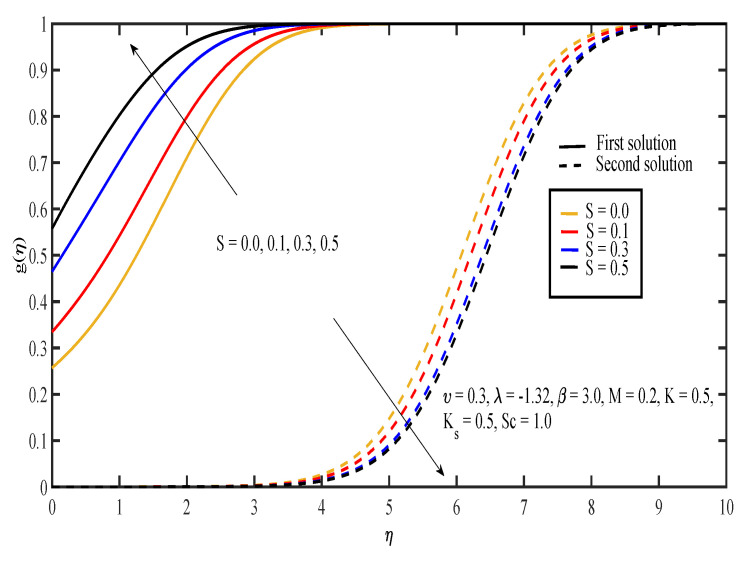
Concentration profiles for different values of suction parameter, *S*.

**Figure 12:**
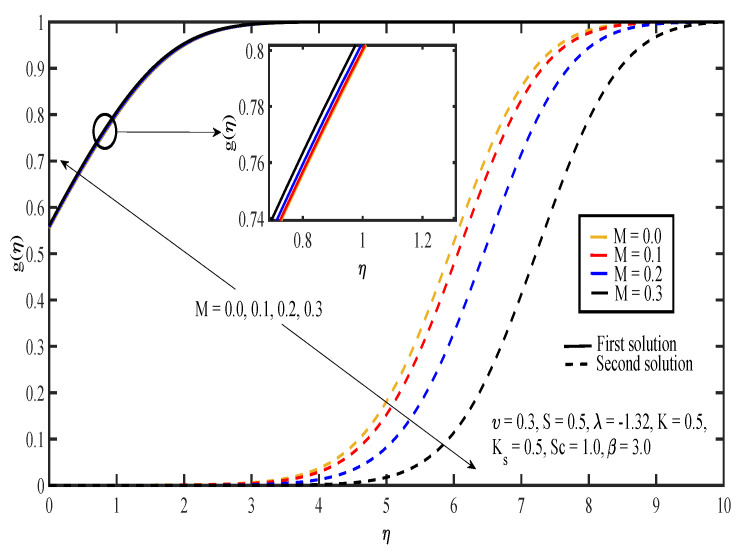
Concentration profiles for different values of magnetic parameter, *M*.

**Figure 13:**
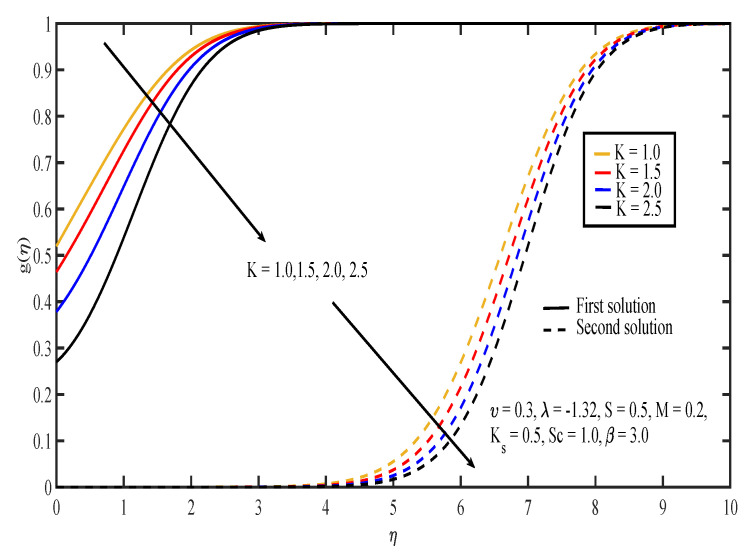
Concentration profiles for different values of the strength of the homogeneous reaction, *K*.

**Figure 14:**
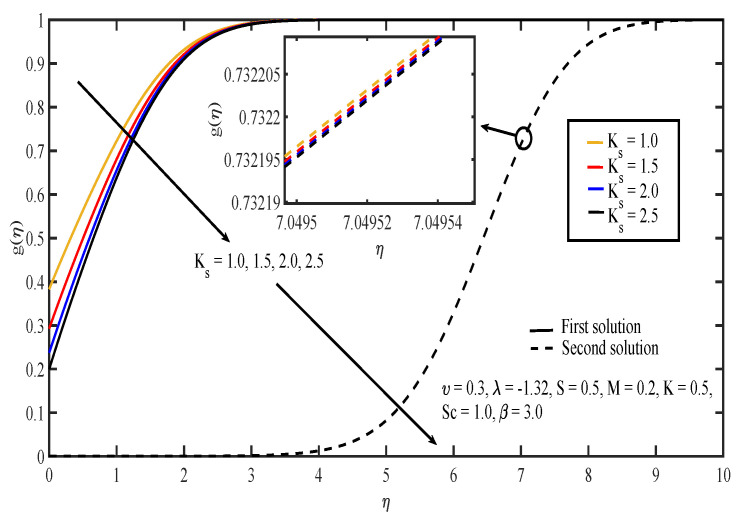
Concentration profiles for different values of the strength of the heterogeneous reaction, $K_{s}$.

**Figure 15:**
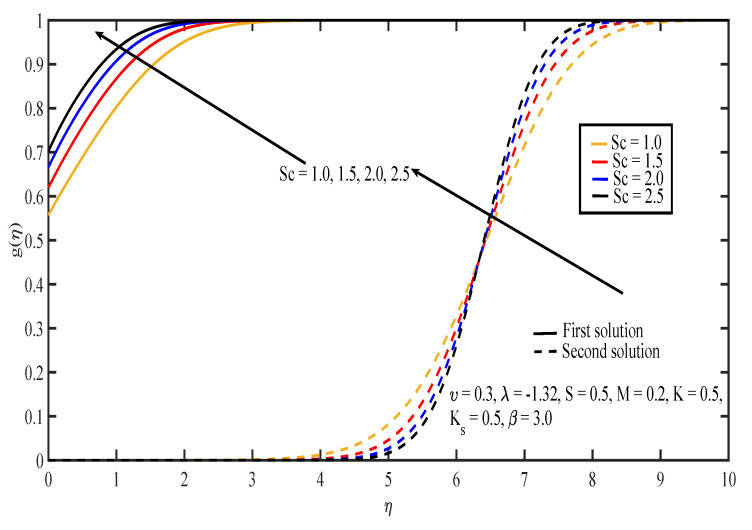
Concentration profiles for different values of Schmidt number, Sc.

**Figure 16:**
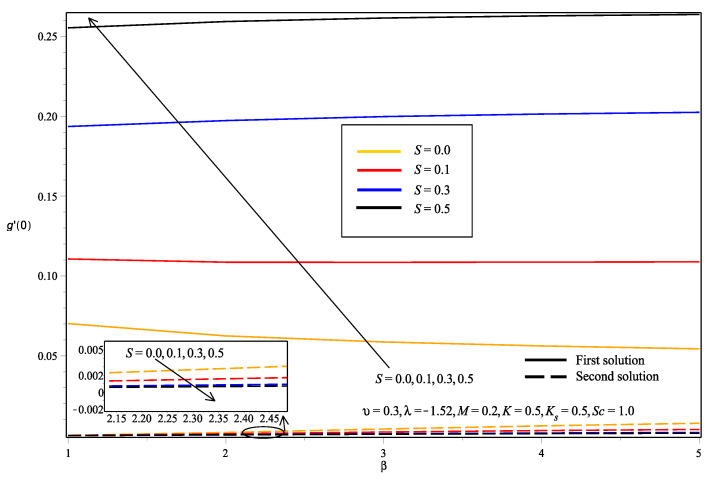
Effects of suction parameter, *S*, on the concentration gradient at the surface, $g^{\prime }\left(0\right)$, with Casson parameter, β.

The effects of various values of parameters on the concentration, g(η), are illustrated in Figure 8, Figure 9, Figure 10, Figure 11, Figure 12, Figure 13, Figure 14 and Figure 15. Figure 8 illustrates the effect of λ on g(η). The concentration decreased as |λ| increased in the first solution, whereas the concentration increased as |λ| increased in the second solution. Then, Figure 9 displays that the concentration profiles for both solutions increased as β increased. The thickness of concentration boundary layer reduced in both solutions because of the increasing elasticity stress parameter. In Figure 10, the concentration for the first solution increased as slip parameter, υ, increased, which was contrary to the second solution. Sheikh and Abbas [20] stated that the concentration increased when the slip parameter increased due to the reduction of available area in the boundary layer, which resulted from the shrinking of the sheet. The same behavior is observed in Figure 11 and Figure 12, where the increase in *S* and *M* caused g(η) to increase in the first solution and decrease in the second solution. The thickness of the boundary layer for the first solution became smaller as *S* and *M* increased. Besides that, the homogeneous-heterogeneous reactions effects on g(η) are depicted in Figure 13 and Figure 14. It can be noted in both figures that the concentration decreased as *K* and $K_{s}$ increased. This was because the reaction rate increased as the values of *K* and $K_{s}$ increased, which caused the concentration of the species to decrease. The Schmidt number, Sc, is the ratio of the viscous diffusion rate to the molecular diffusion rate. When the value of Sc>1, the rate of viscous diffusion was higher than the rate of molecular diffusion. This caused the fluid velocity to decrease and the concentration of reactants to increase, as shown in Figure 15. Last but not least, Figure 16 shows that the presence of *S* caused the concentration gradient at the surface, $g^{\prime }\left(0\right)$, to increase as β increased.

Since the solutions obtained in this paper were of dual solutions, stability analysis was done for the solutions. The smallest eigenvalue, γ, was calculated by performing the analysis using the bvp4c solver in MATLAB. The positive value of γ implied stable flow, while the negative value of γ implied unstable flow. Table 3 shows the values of γ obtained when $K=K_{s}=0.5$, Sc=1.0 and M=0.2. It can be observed that the values of γ were approaching zero as λ approached the critical value, $\lambda_{c}$. Besides that, it can be noted that γ>0 for the first solution and γ<0 for the second solution. Therefore, the first solution is said to be stable and physically meaningful. This agrees with the above discussions. The results of the first solution seemed to be more fitted with the physical explanation provided from previous studies.

## 4. Conclusions

A study on homogeneous-heterogeneous reactions on MHD flow of Casson fluid was carried out. A shrinking sheet in the presence of suction and slip effects was considered as the boundary. Similarity transformations were introduced to convert the partial differential equations into ordinary differential equations. The bvp4c solver was then used to solve the transformed equations and boundary conditions. The results showed that dual solutions existed at certain ranges of parameters. The results were presented in the form of tables and graphs. The effect of various parameters on fluid velocity and concentration were analyzed and discussed. Since the problem contained two solutions, stability analysis was done to determine the stability of the solutions. The values of the smallest eigenvalue were computed using the bvp4c solver and presented in a table. Based on the results, the first solution was stable and physically meaningful. Then, the following were concluded based on the findings of the first solution:

1. The skin friction coefficient increased as suction increased, which indicates that the wall shear stress increased with increasing suction.
2. The skin friction coefficient decreased as the magnitude of the shrinking parameter increased.
3. The fluid velocity and concentration decreased as the value of the shrinking parameter increased.
4. The fluid velocity and concentration increased in the presence of the magnetic parameter.
5. The fluid velocity and concentration increased as the value of the Casson parameter, slip parameter and suction increased.
6. The stronger the strength of homogeneous-heterogeneous reactions, the lower the concentration.
7. The bigger the value of the Schmidt number, the higher the concentration.

## Figures and Tables

**Table 1:** Comparison of the values of local skin friction coefficient, $f^{\prime \prime }\left(0\right)$, for various values of λ when $K=K_{s}=0.5$, Sc=1.0, M=S=υ=0.0 and $\beta =10^{8}$.

λ	Present Study		Kameswaran et al. [31]		Bhattacharyya [32]	
	First Solution	Second Solution	First Solution	Second Solution	First Solution	Second Solution
−1.00	1.3288168	0	1.3288169	0	1.3288169	0
−1.15	1.0822311	0.1167021	1.0822312	0.1167021	1.0822316	0.1167023
−1.20	0.9324733	0.2336497	0.9324734	0.2336497	0.9324728	0.2336491
−1.2465	0.5842814	0.5542963	0.5842817	0.5542962	0.5842915	0.5542856

**Table 2:** Values of the local skin friction coefficient $f^{\prime \prime }\left(0\right)$ for various values of *S* and λ when K=0.5, $K_{s}=0.5$, Sc=1.0, M=0.2, β=3.0 and υ=0.3.

*S*	λ	$f^{\prime \prime }\left(0\right)$	
		First Solution	Second Solution
0.0	−1.54	1.098778769	0.680183640
	−1.55	1.066176015	0.725484949
	−1.56	1.020480243	0.783972152
0.1	−1.60	1.201480040	0.720639471
	−1.62	1.140272080	0.808669739
	−1.63	1.090694586	0.871784903
0.3	−1.74	1.401462735	0.841612630
	−1.76	1.352236872	0.920337136
	−1.78	1.269663918	1.032688556
0.5	−1.92	1.576816297	1.060694430
	−1.94	1.515092506	1.154690179
	−1.95	1.465920061	1.220084931

**Table 3:** Smallest eigenvalues γ for different values of λ when K=0.5, $K_{s}=0.5$, Sc=1.0 and M=0.2.

*S*	λ	γ	
		First Solution	Second Solution
0	−1.567	0.11120	−0.10946
	−1.569	0.03000	−0.02988
	−1.5691	0.01824	−0.01819
0.1	−1.635	0.12316	−0.12112
	−1.637	0.05977	−0.05929
	−1.6376	0.01136	−0.01134
0.3	−1.785	0.14267	−0.14016
	−1.788	0.05146	−0.05113
	−1.7884	0.01772	−0.01768
0.5	−1.955	0.14708	−0.14462
	−1.958	0.06012	−0.05971
	−1.9586	0.00710	−0.00710

## Abbreviations

A,B	Chemical species	
a,b	Concentrations of chemical species *A* and *B*, respectively	
$a_{0}$	Uniform concentration of *A*	
$C_{f}$	Dimensionless skin friction coefficient	
c,m	Constant	
$B_{0}$	Strength of the magnetic field	
$D_{A},D_{B}$	Diffusion coefficient for species *A* and *B*, respectively	
$f^{\prime \prime }\left(0\right)$	Local skin friction coefficient	
$f^{\prime }\left(\eta \right)$	Fluid velocity	
$g^{\prime }\left(0\right)$	Concentration gradient	
g(η)	Concentration	
$k_{c},k_{s}$	Rate constants	
*K*	Strength of homogeneous reaction	
$K_{s}$	Strength of heterogeneous reaction	
*L*	Velocity slip length parameter	m
*M*	Magnetic parameter	
MHD	Magnetohydrodynamics	
$p_{y}$	Yield stress	kg/(m·s${}^{2}$)
Re	Reynolds number	
*S*	Suction parameter	
Sc	Schmidt number	
*t*	Time	s
$u_{e}$	Velocity of external flows	m/s
$u_{w}$	Velocity of the sheet along the *x*-direction	m/s
u,v	Velocity components in the *x*- and *y*-directions, respectively	m/s
$v_{w}$	Constant mass transfer velocity	m/s
η	Similarity variable	
δ	Ratio of diffusion coefficients	
$\mu_{B}$	Plastic dynamic viscosity of Casson fluid	kg/(m·s)
λ	Shrinking parameter	
β	Casson parameter	
υ	Velocity slip parameter	
$\lambda_{c}$	Critical point	
γ	Smallest eigenvalue	
ν	Kinematic viscosity	$m^{2}/s$
σ	Electrical conductivity	s${}^{3}$A${}^{2}$/(kg ·m${}^{3}$)
τ	Dimensionless time variable	
ρ	Density	${\mathrm{kg}/m}^{3}$
$\pi_{c}$	Critical value for the product of the component of the deformation rate with itself	1/s

## Appendix A. Derivation of the Problem

The governing partial differential equations of the problem are:

(A1) $$\begin{array}{cc} \frac{\partial u}{\partial x}+\frac{\partial v}{\partial y} & =0, \end{array}$$

(A2) $$\begin{array}{cc} u\frac{\partial u}{\partial x}+v\frac{\partial u}{\partial y} & =u_{e}\frac{du_{e}}{dx}+\nu \left(1+\frac{1}{\beta }\right)\frac{\partial^{2}u}{\partial y^{2}}+\frac{\sigma B_{0}^{2}}{\rho }\left(u_{e}-u\right), \end{array}$$

(A3) $$\begin{array}{cc} u\frac{\partial a}{\partial x}+v\frac{\partial a}{\partial y} & =D_{A}\frac{\partial^{2}a}{\partial y^{2}}-k_{c}ab^{2}, \end{array}$$

(A4) $$\begin{array}{cc} u\frac{\partial b}{\partial x}+v\frac{\partial b}{\partial y} & =D_{B}\frac{\partial^{2}b}{\partial y^{2}}+k_{c}ab^{2}, \end{array}$$

with the boundary conditions:

(A5) $$\begin{array}{c} u\left(0\right)=u_{w}\left(x\right)=mx+L\left(1+\frac{1}{\beta }\right)\frac{\partial u}{\partial y},\;\;\;\;\;v\left(0\right)=-v_{w},\\ D_{A}\frac{\partial a}{\partial y}{|}_{y=0}=k_{s}a\left(0\right),\;\;\;\;\;D_{B}\frac{\partial b}{\partial y}{|}_{y=0}=-k_{s}a\left(0\right),\\ u\left(\infty \right)=u_{e}\left(x\right)=cx,\;\;\;\;\;a\left(\infty \right)=a_{0},\;\;\;\;\;b\left(\infty \right)=0, \end{array}$$

Then, the following dimensionless variables are introduced:

(A6) $$\begin{array}{c} \eta =\sqrt{\frac{c}{\nu }}y,\;\;\;\;\;u=cxf^{\prime }\left(\eta \right),\;\;\;\;\;v=-\sqrt{c\nu }f\left(\eta \right),\;\;\;\;\;g\left(\eta \right)=\frac{a}{a_{0}},\;\;\;\;\;h\left(\eta \right)=\frac{b}{a_{0}}, \end{array}$$

for similarity transformations of the governing equations.

From (A6),

(A7) $$\begin{array}{cc} u & =cxf^{\prime }\left(\eta \right),\\ \frac{\partial u}{\partial x} & =cf^{\prime }\left(\eta \right), \end{array}$$

(A8) $$\begin{array}{cc} \frac{\partial u}{\partial y} & =c\sqrt{\frac{c}{\nu }}xf^{\prime \prime }\left(\eta \right), \end{array}$$

(A9) $$\begin{array}{cc} \frac{\partial^{2}u}{\partial y^{2}} & =c\sqrt{\frac{c}{\nu }}\sqrt{\frac{c}{\nu }}xf^{\prime \prime \prime }\left(\eta \right)\\ & =\frac{c^{2}}{\nu }xf^{\prime \prime \prime }\left(\eta \right). \end{array}$$

(A10) $$\begin{array}{cc} v & =-\sqrt{c\nu }f\left(\eta \right),\\ \frac{\partial v}{\partial y} & =-\sqrt{c\nu }\sqrt{\frac{c}{\nu }}f^{\prime }\left(\eta \right)\\ & =-cf^{\prime }\left(\eta \right). \end{array}$$

(A11) $$\begin{array}{cc} a & =a_{0}g\left(\eta \right), \end{array}$$

(A12) $$\begin{array}{cc} \frac{\partial a}{\partial x} & =0, \end{array}$$

(A13) $$\begin{array}{cc} \frac{\partial a}{\partial y} & =a_{0}\sqrt{\frac{c}{\nu }}g^{\prime }\left(\eta \right), \end{array}$$

(A14) $$\begin{array}{cc} \frac{\partial^{2}a}{\partial y^{2}} & =a_{0}\sqrt{\frac{c}{\nu }}\sqrt{\frac{c}{\nu }}g^{\prime \prime }\left(\eta \right)\\ & =\frac{a_{0}c}{\nu }g^{\prime \prime }\left(\eta \right). \end{array}$$

(A15) $$\begin{array}{cc} b & =a_{0}h\left(\eta \right), \end{array}$$

(A16) $$\begin{array}{cc} \frac{\partial b}{\partial x} & =0, \end{array}$$

(A17) $$\begin{array}{cc} \frac{\partial b}{\partial y} & =a_{0}\sqrt{\frac{c}{\nu }}h^{\prime }\left(\eta \right), \end{array}$$

(A18) $$\begin{array}{cc} \frac{\partial^{2}b}{\partial y^{2}} & =a_{0}\sqrt{\frac{c}{\nu }}\sqrt{\frac{c}{\nu }}h^{\prime \prime }\left(\eta \right)\\ & =\frac{a_{0}c}{\nu }h^{\prime \prime }\left(\eta \right). \end{array}$$

(A19) $$\begin{array}{cc} u_{e} & =cx, \end{array}$$

(A20) $$\begin{array}{cc} \frac{du_{e}}{dx} & =c. \end{array}$$

### Appendix A.1. Derivation of the Ordinary Differential Equations

Substitute Equations (A7) and (A10) into Equation (A1) to obtain,

$$\begin{array}{cc} \frac{\partial u}{\partial x}+\frac{\partial v}{\partial y} & =0\\ cf^{\prime }\left(\eta \right)+\left(-cf^{\prime }\left(\eta \right)\right) & =0. \end{array}$$

Thus, this satisfies the continuity equation.

From Equation (A2),

$$\begin{array}{cc} u\frac{\partial u}{\partial x}+v\frac{\partial u}{\partial y} & =u_{e}\frac{du_{e}}{dx}+\nu \left(1+\frac{1}{\beta }\right)\frac{\partial^{2}u}{\partial y^{2}}+\frac{\sigma B_{0}^{2}}{\rho }\left(u_{e}-u\right), \end{array}$$

substitute Equations (A6)–(A9), (A19) and (A20) into the above equation to obtain,

$$\begin{array}{cc} \left(cxf^{\prime }\right)\left(cf^{\prime }\left(\eta \right)\right)+\left(-\sqrt{c\nu }f\left(\eta \right)\right)\left(c\sqrt{\frac{c}{\nu }}xf^{\prime \prime }\left(\eta \right)\right) & =\left(cx\right)\left(c\right)+\nu \left(1+\frac{1}{\beta }\right)\left(\frac{c^{2}}{\nu }xf^{\prime \prime \prime }\left(\eta \right)\right)\\ & \;\;\;\;\;\;\;\;+\frac{\sigma B_{0}^{2}}{\rho }\left(cx-cxf^{\prime }\left(\eta \right)\right)\\ c^{2}x{\left[f^{\prime }\left(\eta \right)\right]}^{2}-c^{2}xf\left(\eta \right)f^{\prime \prime }\left(\eta \right) & =c^{2}x+\left(1+\frac{1}{\beta }\right)\left(c^{2}xf^{\prime \prime \prime }\left(\eta \right)\right)+\frac{\sigma B_{0}^{2}}{\rho }cx\left(1-f^{\prime }\left(\eta \right)\right), \end{array}$$

then multiply the equation with $\frac{1}{c^{2}x}$ to form:

(A21) $$\begin{array}{cc} \left(1+\frac{1}{\beta }\right)f^{\prime \prime \prime }+ff^{\prime \prime }-f^{\prime 2}+M^{2}\left(1-f^{\prime }\right)+1 & =0, \end{array}$$

where $M^{2}=\frac{\sigma B_{0}^{2}}{\rho c}$.

Next, from Equation (A3),

$$\begin{array}{cc} u\frac{\partial a}{\partial x}+v\frac{\partial a}{\partial y} & =D_{A}\frac{\partial^{2}a}{\partial y^{2}}-k_{c}ab^{2}, \end{array}$$

substitute Equations (A6) and (A11)–(A14) into the above equation to obtain,

$$\begin{array}{cc} \left(cxf^{\prime }\left(\eta \right)\right)\left(0\right)+\left(-\sqrt{c\nu }f\left(\eta \right)\right)\left(a_{0}\sqrt{\frac{c}{\nu }}g^{\prime }\left(\eta \right)\right) & =D_{A}\left(\frac{a_{0}c}{\nu }g^{\prime \prime }\left(\eta \right)\right)-k_{c}\left(a_{0}g\left(\eta \right)\right){\left(a_{0}h\left(\eta \right)\right)}^{2}\\ -ca_{0}f\left(\eta \right)g^{\prime }\left(\eta \right) & =D_{A}\frac{a_{0}c}{\nu }g^{\prime \prime }\left(\eta \right)-k_{c}a_{0}^{3}g\left(\eta \right){\left[h\left(\eta \right)\right]}^{2}, \end{array}$$

then multiply the equation with $\frac{1}{a_{0}c}$ to form:

(A22) $$\begin{array}{cc} -f\left(\eta \right)g^{\prime }\left(\eta \right) & =\frac{D_{A}}{\nu }g^{\prime \prime }\left(\eta \right)-\frac{k_{c}a_{0}^{2}}{c}g\left(\eta \right){\left[h\left(\eta \right)\right]}^{2}\\ \frac{1}{Sc}g^{\prime \prime }+fg^{\prime }-Kgh^{2}=0, \end{array}$$

where $Sc=\frac{\nu }{D_{A}}$ and $K=\frac{k_{c}a_{0}^{2}}{c}$.

Last but not least, from Equation (A4),

$$\begin{array}{cc} u\frac{\partial b}{\partial x}+v\frac{\partial b}{\partial y} & =D_{B}\frac{\partial^{2}b}{\partial y^{2}}+k_{c}ab^{2}, \end{array}$$

substitute Equations (A6) and (A15)–(A18) into the above equation to obtain,

$$\begin{array}{cc} \left(cxf^{\prime }\left(\eta \right)\right)\left(0\right)+\left(-\sqrt{c\nu }f\left(\eta \right)\right)\left(a_{0}\sqrt{\frac{c}{\nu }}h^{\prime }\left(\eta \right)\right) & =D_{B}\left(\frac{a_{0}c}{\nu }h^{\prime \prime }\left(\eta \right)\right)+k_{c}\left(a_{0}g\left(\eta \right)\right){\left(a_{0}h\left(\eta \right)\right)}^{2}\\ -ca_{0}f\left(\eta \right)h^{\prime }\left(\eta \right) & =D_{B}\frac{a_{0}c}{\nu }h^{\prime \prime }\left(\eta \right)+k_{c}a_{0}^{3}g\left(\eta \right){\left[h\left(\eta \right)\right]}^{2}, \end{array}$$

then multiply the equation with $\frac{1}{a_{0}c}$ to form:

(A23) $$\begin{array}{cc} -f\left(\eta \right)h^{\prime }\left(\eta \right) & =\frac{D_{B}}{\nu }h^{\prime \prime }\left(\eta \right)+\frac{k_{c}a_{0}^{2}}{c}g\left(\eta \right){\left[h\left(\eta \right)\right]}^{2}\\ \frac{\delta }{Sc}h^{\prime \prime }+fh^{\prime }+Kgh^{2} & =0, \end{array}$$

where $\delta =\frac{D_{B}}{D_{A}}$.

By taking the assumption of $D_{A}$ equal to $D_{B}$, δ is equal to one. Therefore, Equation (A22) is equal to Equation (A23). These equations are then reduced to:

(A24) $$\begin{array}{c} \frac{1}{Sc}g^{\prime \prime }+fg^{\prime }-Kg{\left(1-g\right)}^{2}=0. \end{array}$$

### Appendix A.2. Derivation of Boundary Conditions

The boundary conditions are given as:

(A25) $$\begin{array}{c} u\left(0\right)=u_{w}\left(x\right)=mx+L\left(1+\frac{1}{\beta }\right)\frac{\partial u}{\partial y},\;\;\;\;\;v\left(0\right)=-v_{w},\\ D_{A}\frac{\partial a}{\partial y}{|}_{y=0}=k_{s}a\left(0\right),\;\;\;\;\;D_{B}\frac{\partial b}{\partial y}{|}_{y=0}=-k_{s}a\left(0\right),\\ u\left(\infty \right)=u_{e}\left(x\right)=cx,\;\;\;\;\;a\left(\infty \right)=a_{0},\;\;\;\;\;b\left(\infty \right)=0. \end{array}$$

In (A6), when y=0, η=0. Thus, from (A25),

$$\begin{array}{cc} u(0) & =u_{w}\left(x\right)\\ & =mx+L\left(1+\frac{1}{\beta }\right)\frac{\partial u}{\partial y}, \end{array}$$

substitute Equations (A6) and (A8) into the above equation to obtain,

$$\begin{array}{cc} cxf^{\prime }\left(0\right) & =mx+L\left(1+\frac{1}{\beta }\right)\left(c\sqrt{\frac{c}{\nu }}xf^{\prime \prime }(0\right)\\ f^{\prime }\left(0\right) & =\frac{m}{c}+L\left(1+\frac{1}{\beta }\right)\left(\sqrt{\frac{c}{\nu }}f^{\prime \prime }\left(0\right)\right)\\ f^{\prime }\left(0\right) & =\lambda +\upsilon \left(1+\frac{1}{\beta }\right)f^{\prime \prime }\left(0\right), \end{array}$$

where $\lambda =\frac{m}{c}$ and $\upsilon =L\sqrt{\frac{c}{\nu }}$.

$$\begin{array}{cc} v(0) & =-v_{w}, \end{array}$$

substitute (A6) into the above equation to obtain:

$$\begin{array}{cc} -\sqrt{c\nu }f\left(0\right) & =-v_{w},\\ f(0) & =\frac{v_{w}}{\sqrt{c\nu }},\\ f(0) & =S, \end{array}$$

where $S=\frac{v_{w}}{\sqrt{c\nu }}$.

$$\begin{array}{cc} D_{A}\frac{\partial a}{\partial y}{|}_{y=0} & =k_{s}a\left(0\right), \end{array}$$

substitute Equations (A11) and (A13) into the equation to obtain:

(A26) $$\begin{array}{cc} D_{A}\left(a_{0}\sqrt{\frac{c}{\nu }}g^{\prime }\left(0\right)\right) & =k_{s}\left(a_{0}g\left(0\right)\right)\\ D_{A}\sqrt{\frac{c}{\nu }}g^{\prime }\left(0\right) & =k_{s}g\left(0\right)\\ g^{\prime }\left(0\right) & =K_{s}g\left(0\right), \end{array}$$

where $K_{s}=\frac{k_{s}Re^{-1/2}}{D_{A}}$ and $Re=\frac{c}{\nu }$.

$$\begin{array}{cc} D_{B}\frac{\partial b}{\partial y}{|}_{y=0} & =-k_{s}a\left(0\right), \end{array}$$

substitute Equations (A11) and (A17) into the equation to obtain,

(A27) $$\begin{array}{cc} D_{B}\left(a_{0}\sqrt{\frac{c}{\nu }}h^{\prime }\left(0\right)\right) & =-k_{s}\left(a_{0}g\left(0\right)\right)\\ D_{B}\sqrt{\frac{c}{\nu }}h^{\prime }\left(0\right) & =k_{s}g\left(0\right)\\ D_{B}h^{\prime }\left(0\right) & =-k_{s}\sqrt{\frac{\nu }{c}}g\left(0\right)\\ \delta h^{\prime }\left(0\right) & =-K_{s}g\left(0\right). \end{array}$$

In (A6), as y→∞, η→∞. Thus, from: (A25),

$$\begin{array}{cc} u(\infty ) & =u_{e}\left(x\right)\\ & =cx, \end{array}$$

substitute (A6) into the equation to obtain,

$$\begin{array}{cc} cxf^{\prime }\left(\infty \right) & =cx\\ f^{\prime }\left(\infty \right) & =1.\\ a(\infty ) & =a_{0}, \end{array}$$

substitute Equation (A11) into the above equation to obtain,

(A28) $$\begin{array}{cc} a_{0}g\left(\infty \right) & =a_{0}\\ g(\infty ) & =1. \end{array}$$

$$\begin{array}{cc} b(\infty ) & =0, \end{array}$$

substitute Equation (15) into the above equation to obtain,

(A29) $$\begin{array}{cc} a_{0}h\left(\infty \right) & =0\\ h(\infty ) & =0. \end{array}$$

By taking the assumption of $D_{A}$ equal to $D_{B}$, δ is equal to one. Therefore, Equation (A26) is equal to Equation (A27), and Equation (A28) is equal to Equation (A29). These equations can be reduced to:

$$\begin{array}{c} g^{\prime }\left(0\right)=K_{s}g\left(0\right),\;\;\;\;\;g\left(\infty \right)=1. \end{array}$$

## Appendix B. Derivation for Stability Analysis

The unsteady state of the problem can be written as:

(A30) $$\begin{array}{cc} \frac{\partial u}{\partial t}+u\frac{\partial u}{\partial x}+v\frac{\partial u}{\partial y} & =u_{e}\frac{\partial u_{e}}{\partial x}+\nu \left(1+\frac{1}{\beta }\right)\frac{\partial^{2}u}{\partial y^{2}}+\frac{\sigma B_{0}^{2}}{\rho }\left(u_{e}-u\right), \end{array}$$

(A31) $$\begin{array}{cc} \frac{\partial a}{\partial t}+u\frac{\partial a}{\partial x}+v\frac{\partial a}{\partial y} & =D_{A}\frac{\partial^{2}a}{\partial y^{2}}-k_{c}ab^{2}, \end{array}$$

(A32) $$\begin{array}{cc} \frac{\partial b}{\partial t}+u\frac{\partial b}{\partial x}+v\frac{\partial b}{\partial y} & =D_{B}\frac{\partial^{2}b}{\partial y^{2}}+k_{c}ab^{2}, \end{array}$$

with new dimensionless variables:

(A33) $$\begin{array}{c} \begin{array}{cc} \eta =\sqrt{\frac{c}{\nu }}y,\;\;\;\;\;u=cxf^{\prime }\left(\eta ,\tau \right),\;\;\;\;\;v & =-\sqrt{c\nu }f\left(\eta ,\tau \right),\;\;\;\;\;g\left(\eta ,\tau \right)=\frac{a}{a_{0}},\\ h(\eta ,\tau ) & =\frac{b}{a_{0}},\;\;\;\;\;\;\tau =ct. \end{array} \end{array}$$

From (A33),

$$\begin{array}{cc} u & =cx\frac{\partial f}{\partial \eta }, \end{array}$$

(A34) $$\begin{array}{cc} \frac{\partial u}{\partial x} & =c\frac{\partial f}{\partial \eta }, \end{array}$$

(A35) $$\begin{array}{cc} \frac{\partial u}{\partial y} & =c\sqrt{\frac{c}{\nu }}x\frac{\partial^{2}f}{\partial \eta^{2}}, \end{array}$$

(A36) $$\begin{array}{cc} \frac{\partial^{2}u}{\partial y^{2}} & =\frac{c^{2}}{\nu }x\frac{\partial^{3}f}{\partial \eta^{3}}, \end{array}$$

(A37) $$\begin{array}{cc} \frac{\partial u}{\partial t} & =c^{2}x\frac{\partial^{2}f}{\partial \eta \partial \tau }. \end{array}$$

(A38) $$\begin{array}{cc} g(\eta ,\tau ) & =\frac{a}{a_{0}},\\ a & =a_{0}g\left(\eta ,\tau \right), \end{array}$$

(A39) $$\begin{array}{cc} \frac{\partial a}{\partial x} & =0, \end{array}$$

(A40) $$\begin{array}{cc} \frac{\partial a}{\partial y} & =a_{0}\sqrt{\frac{c}{\nu }}\frac{\partial g}{\partial \eta }, \end{array}$$

(A41) $$\begin{array}{cc} \frac{\partial^{2}a}{\partial y^{2}} & =\frac{a_{0}c}{\nu }\frac{\partial^{2}g}{\partial \eta^{2}}, \end{array}$$

(A42) $$\begin{array}{cc} \frac{\partial a}{\partial t} & =a_{0}c\frac{\partial g}{\partial \tau }. \end{array}$$

(A43) $$\begin{array}{cc} h(\eta ,\tau ) & =\frac{b}{a_{0}},\\ b & =a_{0}h\left(\eta ,\tau \right), \end{array}$$

(A44) $$\begin{array}{cc} \frac{\partial b}{\partial x} & =0, \end{array}$$

(A45) $$\begin{array}{cc} \frac{\partial b}{\partial y} & =a_{0}\sqrt{\frac{c}{\nu }}\frac{\partial h}{\partial \eta }, \end{array}$$

(A46) $$\begin{array}{cc} \frac{\partial^{2}b}{\partial y^{2}} & =\frac{a_{0}c}{\nu }\frac{\partial^{2}h}{\partial \eta^{2}}, \end{array}$$

(A47) $$\begin{array}{cc} \frac{\partial b}{\partial t} & =a_{0}c\frac{\partial h}{\partial \tau }. \end{array}$$

(A48) $$\begin{array}{cc} u_{e} & =cx, \end{array}$$

(A49) $$\begin{array}{cc} \frac{\partial u_{e}}{\partial x} & =c. \end{array}$$

Substitute Equations (A33)–(A49) into Equations (A30)–(A32) to obtain the following equations:

From Equation (A30),

$$\begin{array}{cc} \frac{\partial u}{\partial t}+u\frac{\partial u}{\partial x}+v\frac{\partial u}{\partial y} & =u_{e}\frac{\partial u_{e}}{\partial x}+\nu \left(1+\frac{1}{\beta }\right)\frac{\partial^{2}u}{\partial y^{2}}\\ & \;\;\;\;\;\;\;\;+\frac{\sigma B_{0}^{2}}{\rho }\left(u_{e}-u\right) \end{array}$$

$$\begin{array}{cc} \left(c^{2}x\frac{\partial^{2}f}{\partial \eta \partial \tau }\right)+\left(cx\frac{\partial f}{\partial \eta }\right)\left(c\frac{\partial f}{\partial \eta }\right)+\left(-\sqrt{c\nu }f\right)\left(c\sqrt{\frac{c}{\nu }}x\frac{\partial^{2}f}{\partial \eta^{2}}\right) & =cx\left(c\right)+\nu \left(1+\frac{1}{\beta }\right)\left(\frac{c^{2}}{\nu }x\frac{\partial^{3}f}{\partial \eta^{3}}\right)\\ & \;\;\;\;\;\;\;\;\;+\frac{\sigma B_{0}^{2}}{\rho }\left(cx-cx\frac{\partial f}{\partial \eta }\right)\\ c^{2}x\frac{\partial^{2}f}{\partial \eta \partial \tau }+c^{2}x{\left(\frac{\partial f}{\partial \eta }\right)}^{2}-c^{2}xf\frac{\partial^{2}f}{\partial \eta^{2}} & =c^{2}x+c^{2}x\left(1+\frac{1}{\beta }\right)\frac{\partial^{3}f}{\partial \eta^{3}}\\ & \;\;\;\;\;\;\;\;+\frac{\sigma B_{0}^{2}cx}{\rho }\left(1-\frac{\partial f}{\partial \eta }\right) \end{array}$$

multiply the equation with $\frac{1}{c^{2}x}$ to obtain,

(A50) $$\begin{array}{cc} \left(1+\frac{1}{\beta }\right)\frac{\partial^{3}f}{\partial \eta^{3}}+f\frac{\partial^{2}f}{\partial \eta^{2}}-{\left(\frac{\partial f}{\partial \eta }\right)}^{2}+M^{2}\left(1-\frac{\partial f}{\partial \eta }\right)+1-\frac{\partial^{2}f}{\partial \eta \partial \tau } & =0. \end{array}$$

From Equation (A31),

$$\begin{array}{cc} \frac{\partial a}{\partial t}+u\frac{\partial a}{\partial x}+v\frac{\partial a}{\partial y} & =D_{A}\frac{\partial^{2}a}{\partial y^{2}}-k_{c}ab^{2}\\ \left(a_{0}c\frac{\partial g}{\partial \tau }\right)+\left(cx\frac{\partial f}{\partial \eta }\right)\left(0\right)+\left(-\sqrt{c\nu }f\right)\left(a_{0}\sqrt{\frac{c}{\nu }}\frac{\partial g}{\partial \eta }\right) & =D_{A}\left(\frac{a_{0}c}{\nu }\frac{\partial^{2}g}{\partial \eta^{2}}\right)-k_{c}\left(a_{0}g\right){\left(a_{0}h\right)}^{2}\\ a_{0}c\frac{\partial g}{\partial \tau }-a_{0}cf\frac{\partial g}{\partial \eta } & =D_{A}\frac{a_{0}c}{\nu }\frac{\partial^{2}g}{\partial \eta^{2}}-k_{c}a_{0}^{3}g{\left[h\right]}^{2} \end{array}$$

multiply the equation with $\frac{1}{a_{0}c}$ to obtain,

(A51) $$\begin{array}{cc} \frac{1}{Sc}\frac{\partial^{2}g}{\partial \eta^{2}}+f\frac{\partial g}{\partial \eta }-Kgh^{2}-\frac{\partial g}{\partial \tau } & =0, \end{array}$$

where $Sc=\frac{\nu }{D_{A}}$ and $K=\frac{k_{c}a_{0}^{2}}{c}$.

From Equation (A32),

$$\begin{array}{cc} \frac{\partial b}{\partial t}+u\frac{\partial b}{\partial x}+v\frac{\partial b}{\partial y} & =D_{B}\frac{\partial^{2}b}{\partial y^{2}}+k_{c}ab^{2}\\ \left(a_{0}c\frac{\partial h}{\partial \tau }\right)+\left(cx\frac{\partial f}{\partial \eta }\right)\left(0\right)+\left(-\sqrt{c\nu }f\right)\left(a_{0}\sqrt{\frac{c}{\nu }}\frac{\partial h}{\partial \eta }\right) & =D_{B}\left(\frac{a_{0}c}{\nu }\frac{\partial^{2}h}{\partial \eta^{2}}\right)+k_{c}\left(a_{0}g\right){\left(a_{0}h\right)}^{2}\\ a_{0}c\frac{\partial h}{\partial \tau }-a_{0}cf\frac{\partial h}{\partial \eta } & =D_{B}\left(\frac{a_{0}c}{\nu }\frac{\partial^{2}h}{\partial \eta^{2}}\right)+k_{c}a_{0}^{3}g{\left[h\right]}^{2} \end{array}$$

multiply the equation with $\frac{1}{a_{0}c}$ to obtain,

(A52) $$\begin{array}{cc} 1-1\frac{\delta }{Sc}\frac{\partial^{2}h}{\partial \eta^{2}}+f\frac{\partial h}{\partial \eta }+Kgh^{2}-\frac{\partial h}{\partial \tau } & =0, \end{array}$$

where $\delta =\frac{D_{A}}{D_{B}}$.

By the assumption of $D_{A}$ equal to $D_{B}$ and δ=1, Equation (A51) = Equation (A52). Thus, the equations are reduced to:

(A53) $$\begin{array}{c} \frac{1}{Sc}\frac{\partial^{2}g}{\partial \eta^{2}}+f\frac{\partial g}{\partial \eta }-Kg{\left(1-g\right)}^{2}-\frac{\partial g}{\partial \tau }=0. \end{array}$$

The boundary conditions become:

(A54) $$\begin{array}{cc} u(0) & =mx+L\left(1+\frac{1}{\beta }\right)\frac{\partial u}{\partial y}\\ cx\frac{\partial f}{\partial \eta } & =mx+L\left(1+\frac{1}{\beta }\right)\left(c\sqrt{\frac{c}{\nu }}x\frac{\partial^{2}f}{\partial \eta^{2}}\right)\\ \frac{\partial f}{\partial \eta }\left(0,\tau \right) & =\lambda +\upsilon \left(1+\frac{1}{\beta }\right)\frac{\partial^{2}f}{\partial \eta^{2}}\left(0,\tau \right), \end{array}$$

where $\lambda =\frac{m}{c}$ and $\upsilon =L\sqrt{\frac{c}{\nu }}$.

(A55) $$\begin{array}{cc} v(0) & =-v_{w}\\ -\sqrt{c\nu }f\left(0,\tau \right) & =-v_{w}\\ f(0,\tau ) & =S, \end{array}$$

where $S=\frac{v_{w}}{\sqrt{c\nu }}$.

(A56) $$\begin{array}{cc} D_{A}\frac{\partial a}{\partial y}{|}_{y=0} & =k_{s}a\left(0\right)\\ D_{A}\left(a_{0}\sqrt{\frac{c}{\nu }}\frac{\partial g}{\partial \eta }\right) & =k_{s}\left(a_{0}g\left(0,\tau \right)\right)\\ \frac{\partial g}{\partial \eta }\left(0,\tau \right) & =K_{s}g\left(0,\tau \right), \end{array}$$

where $K_{s}=\frac{k_{s}Re^{-1/2}}{D_{A}}$ and $Re=\frac{c}{\nu }$.

(A57) $$\begin{array}{cc} D_{B}\frac{\partial b}{\partial y}{|}_{y=0} & =-k_{s}a\left(0\right)\\ D_{B}\left(a_{0}\sqrt{\frac{c}{\nu }}\frac{\partial h}{\partial \eta }\right) & =-k_{s}\left(a_{0}g\left(0,\tau \right)\right)\\ \delta \frac{\partial h}{\partial \eta }\left(0,\tau \right) & =-K_{s}g\left(0,\tau \right). \end{array}$$

(A58) $$\begin{array}{cc} u(\infty ) & =cx\\ cx\frac{\partial f}{\partial \eta } & =cx\\ \frac{\partial f}{\partial \eta }\left(\infty ,\tau \right) & =1. \end{array}$$

(A59) $$\begin{array}{cc} a(\infty ) & =a_{0}\\ a_{0}g\left(\infty ,\tau \right) & =a_{0}\\ g(\infty ,\tau ) & =1. \end{array}$$

(A60) $$\begin{array}{cc} b(\infty ) & =0\\ a_{0}h\left(\infty ,\tau \right) & =0\\ h(\infty ,\tau ) & =0. \end{array}$$

By the assumption of $D_{A}$ equal to $D_{B}$ and δ=1, the boundary conditions (A56) = (A57) and (A59) = (A60). Thus, the boundary conditions are reduced to:

(A61) $$\begin{array}{c} \frac{\partial g}{\partial \eta }\left(0,\tau \right)=K_{s}g\left(0,\tau \right),\;\;\;\;\;g\left(\infty ,\tau \right)=1. \end{array}$$

In order to test the stability of the steady flow solution $f\left(\eta \right)=f_{0}\left(\eta \right)$ and $g\left(\eta \right)=g_{0}\left(\eta \right)$, the following expressions are introduced:

(A62) $$\begin{array}{c} f\left(\eta ,\tau \right)=f_{0}\left(\eta \right)+e^{-\gamma \tau }F\left(\eta ,\tau \right),\\ g\left(\eta ,\tau \right)=g_{0}\left(\eta \right)+e^{-\gamma \tau }G\left(\eta ,\tau \right), \end{array}$$

where,

(A63) $$\begin{array}{cc} \frac{\partial f}{\partial \eta } & =\frac{\partial f_{0}}{\partial \eta }+e^{-\gamma \tau }\frac{\partial F}{\partial \eta }, \end{array}$$

(A64) $$\begin{array}{cc} \frac{\partial^{2}f}{\partial \eta^{2}} & =\frac{\partial^{2}f_{0}}{\partial \eta^{2}}+e^{-\gamma \tau }\frac{\partial^{2}F}{\partial \eta^{2}}, \end{array}$$

(A65) $$\begin{array}{cc} \frac{\partial^{3}f}{\partial \eta^{3}} & =\frac{\partial^{3}f_{0}}{\partial \eta^{3}}+e^{-\gamma \tau }\frac{\partial^{3}F}{\partial \eta^{3}}, \end{array}$$

(A66) $$\begin{array}{cc} \frac{\partial^{2}f}{\partial \eta \partial \tau } & =-\gamma e^{-\gamma \tau }\frac{\partial F}{\partial \eta }+e^{-\gamma \tau }\frac{\partial^{2}F}{\partial \eta \partial \tau }, \end{array}$$

and

(A67) $$\begin{array}{cc} \frac{\partial g}{\partial \eta } & =\frac{\partial g_{0}}{\partial \eta }+e^{-\gamma \tau }\frac{\partial G}{\partial \eta }, \end{array}$$

(A68) $$\begin{array}{cc} \frac{\partial^{2}g}{\partial \eta^{2}} & =\frac{\partial^{2}g_{0}}{\partial \eta^{2}}+e^{-\gamma \tau }\frac{\partial^{2}G}{\partial \eta^{2}}, \end{array}$$

(A69) $$\begin{array}{cc} \frac{\partial g}{\partial \tau } & =-\gamma e^{-\gamma \tau }G+e^{-\gamma \tau }\frac{\partial G}{\partial \tau }. \end{array}$$

Equations (A62)–(A69) are then substituted into Equations (A50) and (A53) to obtain the following linearized equations:

For Equation (A50),

$$\begin{array}{c} \begin{array}{c} \left(1+\frac{1}{\beta }\right)\frac{\partial^{3}f}{\partial \eta^{3}}+f\frac{\partial^{2}f}{\partial \eta^{2}}-{\left(\frac{\partial f}{\partial \eta }\right)}^{2}+M^{2}\left(1-\frac{\partial f}{\partial \eta }\right)+1-\frac{\partial^{2}f}{\partial \eta \partial \tau }=0\\ \left(1+\frac{1}{\beta }\right)\left(\frac{\partial^{3}f_{0}}{\partial \eta^{3}}+e^{-\gamma \tau }\frac{\partial^{3}F}{\partial \eta^{3}}\right)+\left(f_{0}\left(\eta \right)+e^{-\gamma \tau }F\left(\eta ,\tau \right)\right)\left(\frac{\partial^{2}f_{0}}{\partial \eta^{2}}+e^{-\gamma \tau }\frac{\partial^{2}F}{\partial \eta^{2}}\right)-{\left(\frac{\partial f_{0}}{\partial \eta }+e^{-\gamma \tau }\frac{\partial F}{\partial \eta }\right)}^{2}\\ +M^{2}\left[1-\left(\frac{\partial f_{0}}{\partial \eta }+e^{-\gamma \tau }\frac{\partial F}{\partial \eta }\right)\right]+1-\left(-\gamma e^{-\gamma \tau }\frac{\partial F}{\partial \eta }+e^{-\gamma \tau }\frac{\partial^{2}F}{\partial \eta \partial \tau }\right)=0 \end{array} \end{array}$$

$$\begin{array}{c} \left(1+\frac{1}{\beta }\right)\frac{\partial^{3}f_{0}}{\partial \eta^{3}}+f_{0}\frac{\partial^{2}f_{0}}{\partial \eta^{2}}-{\left(\frac{\partial f_{0}}{\partial \eta }\right)}^{2}+M^{2}\left(1-\frac{\partial f_{0}}{\partial \eta }\right)+1+\left(1+\frac{1}{\beta }\right)e^{-\gamma \tau }\frac{\partial^{3}F}{\partial \eta^{3}}+e^{-\gamma \tau }F\left(\eta ,\tau \right)\frac{\partial^{2}f_{0}}{\partial \eta^{2}}\\ +f_{0}e^{-\gamma \tau }\frac{\partial^{2}F}{\partial \eta^{2}}+e^{-2\gamma \tau }F\frac{\partial^{2}F}{\partial \eta^{2}}-2e^{-\gamma \tau }\frac{\partial f_{0}}{\partial \eta }\frac{\partial F}{\partial \eta }-e^{-2\gamma \tau }{\left(\frac{\partial F}{\partial \eta }\right)}^{2}-M^{2}e^{-\gamma \tau }\frac{\partial F}{\partial \eta }+\gamma e^{-\gamma \tau }\frac{\partial F}{\partial \eta }-e^{-\gamma \tau }\frac{\partial^{2}F}{\partial \eta \partial \tau }=0 \end{array}$$

where from Equation (A21), $\left(1+\frac{1}{\beta }\right)\frac{\partial^{3}f_{0}}{\partial \eta^{3}}+f_{0}\frac{\partial^{2}f_{0}}{\partial \eta^{2}}-{\left(\frac{\partial f_{0}}{\partial \eta }\right)}^{2}+M^{2}\left(1-\frac{\partial f_{0}}{\partial \eta }\right)+1=0$, thus

$$\begin{array}{c} \left(1+\frac{1}{\beta }\right)e^{-\gamma \tau }\frac{\partial^{3}F}{\partial \eta^{3}}+e^{-\gamma \tau }F\frac{\partial^{2}f_{0}}{\partial \eta^{2}}+f_{0}e^{-\gamma \tau }\frac{\partial^{2}F}{\partial \eta^{2}}+e^{-2\gamma \tau }F\frac{\partial^{2}F}{\partial \eta^{2}}-2e^{-\gamma \tau }\frac{\partial f_{0}}{\partial \eta }\frac{\partial F}{\partial \eta }-e^{-2\gamma \tau }{\left(\frac{\partial F}{\partial \eta }\right)}^{2}\\ -M^{2}e^{-\gamma \tau }\frac{\partial F}{\partial \eta }+\gamma e^{-\gamma \tau }\frac{\partial F}{\partial \eta }-e^{-\gamma \tau }\frac{\partial^{2}F}{\partial \eta \partial \tau }=0 \end{array}$$

factorize $e^{-\gamma \tau }$ in the equation to obtain:

$$\begin{array}{c} \begin{array}{c} \left(1+\frac{1}{\beta }\right)\frac{\partial^{3}F}{\partial \eta^{3}}+F\frac{\partial^{2}f_{0}}{\partial \eta^{2}}+f_{0}\frac{\partial^{2}F}{\partial \eta^{2}}+e^{-\gamma \tau }F\frac{\partial^{2}F}{\partial \eta^{2}}-2\frac{\partial f_{0}}{\partial \eta }\frac{\partial F}{\partial \eta }-e^{-\gamma \tau }{\left(\frac{\partial F}{\partial \eta }\right)}^{2}\\ -M^{2}\frac{\partial F}{\partial \eta }+\gamma \frac{\partial F}{\partial \eta }-\frac{\partial^{2}F}{\partial \eta \partial \tau }=0 \end{array} \end{array}$$

as τ→∞, $e^{-\gamma \tau }\to 0$, thus:

(A70) $$\begin{array}{c} \left(1+\frac{1}{\beta }\right)\frac{\partial^{3}F}{\partial \eta^{3}}+f_{0}\frac{\partial^{2}F}{\partial \eta^{2}}+F\frac{\partial^{2}f_{0}}{\partial \eta^{2}}-\left(2\frac{\partial f_{0}}{\partial \eta }+M^{2}-\gamma \right)\frac{\partial F}{\partial \eta }-\frac{\partial^{2}F}{\partial \eta \partial \tau }=0. \end{array}$$

For Equation (A53),

$$\begin{array}{c} \begin{array}{c} \frac{1}{Sc}\frac{\partial^{2}g}{\partial \eta^{2}}+f\frac{\partial g}{\partial \eta }-Kg{\left(1-g\right)}^{2}-\frac{\partial g}{\partial \tau }=0\\ \frac{1}{Sc}\left(\frac{\partial^{2}g_{0}}{\partial \eta^{2}}+e^{-\gamma \tau }\frac{\partial^{2}G}{\partial \eta^{2}}\right)+\left(f_{0}\left(\eta \right)+e^{-\gamma \tau }F\left(\eta ,\tau \right)\right)\left(\frac{\partial g_{0}}{\partial \eta }+e^{-\gamma \tau }\frac{\partial G}{\partial \eta }\right)\\ -K\left(g_{0}\left(\eta \right)+e^{-\gamma \tau }G\left(\eta ,\tau \right)\right){\left[1-\left(g_{0}\left(\eta \right)+e^{-\gamma \tau }G\left(\eta ,\tau \right)\right)\right]}^{2}-\left(-\gamma e^{-\gamma \tau }G+e^{-\gamma \tau }\frac{\partial G}{\partial \tau }\right)=0\\ \frac{1}{Sc}\frac{\partial^{2}g_{0}}{\partial \eta^{2}}+f_{0}\frac{\partial g_{0}}{\partial \eta }-Kg_{0}{\left(1-g_{0}\right)}^{2}+\frac{1}{Sc}e^{-\gamma \tau }\frac{\partial^{2}G}{\partial \eta^{2}}+f_{0}e^{-\gamma \tau }\frac{\partial G}{\partial \eta }+e^{-\gamma \tau }F\frac{\partial g_{0}}{\partial \eta }+e^{-2\gamma \tau }F\frac{\partial G}{\partial \eta }\\ -Kg_{0}\left[\left(2g_{0}-2+e^{-\gamma \tau }G\right)e^{-\gamma \tau }G\right]-Ke^{-\gamma \tau }G{\left(1-g_{0}\right)}^{2}-Ke^{-\gamma \tau }G\left[\left(2g_{0}-2+e^{-\gamma \tau }G\right)e^{-\gamma \tau }G\right]\\ +\gamma e^{-\gamma \tau }G-e^{-\gamma \tau }\frac{\partial G}{\partial \tau }=0 \end{array} \end{array}$$

where from Equation (A24), $\frac{1}{Sc}\frac{\partial^{2}g_{0}}{\partial \eta^{2}}+f_{0}\frac{\partial g_{0}}{\partial \eta }-Kg_{0}{\left(1-g_{0}\right)}^{2}=0$, thus:

$$\begin{array}{c} \begin{array}{c} \frac{1}{Sc}e^{-\gamma \tau }\frac{\partial^{2}G}{\partial \eta^{2}}+f_{0}e^{-\gamma \tau }\frac{\partial G}{\partial \eta }+e^{-\gamma \tau }F\frac{\partial g_{0}}{\partial \eta }+e^{-2\gamma \tau }F\frac{\partial G}{\partial \eta }-Kg_{0}\left[\left(2g_{0}-2+e^{-\gamma \tau }G\right)e^{-\gamma \tau }G\right]\\ -Ke^{-\gamma \tau }G{\left(1-g_{0}\right)}^{2}-Ke^{-\gamma \tau }G\left[\left(2g_{0}-2+e^{-\gamma \tau }G\right)e^{-\gamma \tau }G\right]+\gamma e^{-\gamma \tau }G-e^{-\gamma \tau }\frac{\partial G}{\partial \tau }=0 \end{array} \end{array}$$

factorize $e^{-\gamma \tau }$ in the equation to obtain:

$$\begin{array}{c} \begin{array}{c} \frac{1}{Sc}\frac{\partial^{2}G}{\partial \eta^{2}}+f_{0}\frac{\partial G}{\partial \eta }+F\frac{\partial g_{0}}{\partial \eta }+e^{-\gamma \tau }F\frac{\partial G}{\partial \eta }\\ -Kg_{0}\left[\left(2g_{0}-2+e^{-\gamma \tau }G\right)G\right]-KG{\left(1-g_{0}\right)}^{2}-KG\left[\left(2g_{0}-2+e^{-\gamma \tau }G\right)e^{-\gamma \tau }G\right]+\gamma G-\frac{\partial G}{\partial \tau }=0 \end{array} \end{array}$$

as τ→∞, $e^{-\gamma \tau }\to 0$, thus:

(A71) $$\begin{array}{c} \frac{1}{Sc}\frac{\partial^{2}G}{\partial \eta^{2}}+F\frac{\partial g_{0}}{\partial \eta }+f_{0}\frac{\partial G}{\partial \eta }-\left(K-4Kg_{0}+3Kg_{0}^{2}-\gamma \right)G-\frac{\partial G}{\partial \tau }=0. \end{array}$$

The boundary conditions become:

(A72) $$\begin{array}{cc} \frac{\partial f}{\partial \eta }\left(0,\tau \right) & =\lambda +\upsilon \left(1+\frac{1}{\beta }\right)\frac{\partial^{2}f}{\partial \eta^{2}}\left(0,\tau \right)\\ \frac{\partial f_{0}}{\partial \eta }+e^{-\gamma \tau }\frac{\partial F}{\partial \eta } & =\lambda +\upsilon \left(1+\frac{1}{\beta }\right)\left(\frac{\partial^{2}f_{0}}{\partial \eta^{2}}+e^{-\gamma \tau }\frac{\partial^{2}F}{\partial \eta^{2}}\right)\\ \lambda +\upsilon \left(1+\frac{1}{\beta }\right)\frac{\partial^{2}f_{0}}{\partial \eta^{2}}+e^{-\gamma \tau }\frac{\partial F}{\partial \eta } & =\lambda +\upsilon \left(1+\frac{1}{\beta }\right)\left(\frac{\partial^{2}f_{0}}{\partial \eta^{2}}+e^{-\gamma \tau }\frac{\partial^{2}F}{\partial \eta^{2}}\right)\\ \frac{\partial F}{\partial \eta }\left(0,\tau \right) & =\upsilon \left(1+\frac{1}{\beta }\right)\frac{\partial^{2}F}{\partial \eta^{2}}\left(0,\tau \right). \end{array}$$

(A73) $$\begin{array}{cc} f(0,\tau ) & =S\\ f_{0}\left(0\right)+e^{-\gamma \tau }F\left(0,\tau \right) & =S\\ S+e^{-\gamma \tau }F\left(0,\tau \right) & =S\\ F(0,\tau ) & =0. \end{array}$$

(A74) $$\begin{array}{cc} \frac{\partial g}{\partial \eta }\left(0,\tau \right) & =K_{s}g\left(0,\tau \right)\\ \frac{\partial g_{0}}{\partial \eta }+e^{-\gamma \tau }\frac{\partial G}{\partial \eta } & =K_{s}\left[g_{0}\left(0\right)+e^{-\gamma \tau }G\left(0,\tau \right)\right]\\ K_{s}g_{0}\left(0\right)+e^{-\gamma \tau }\frac{\partial G}{\partial \eta } & =K_{s}\left[g_{0}\left(0\right)+e^{-\gamma \tau }G\left(0,\tau \right)\right]\\ \frac{\partial G}{\partial \eta }\left(0,\tau \right)=K_{s}G\left(0,\tau \right). \end{array}$$

(A75) $$\begin{array}{cc} \frac{\partial f}{\partial \eta }\left(\infty ,\tau \right) & =1\\ \frac{\partial f_{0}}{\partial \eta }+e^{-\gamma \tau }\frac{\partial F}{\partial \eta } & =1\\ 1+e^{-\gamma \tau }\frac{\partial F}{\partial \eta } & =1\\ \frac{\partial F}{\partial \eta }\left(\eta ,\tau \right) & \to 0\;\;\;\;\;\;\;\;\mathrm{as}\;\eta \to \infty . \end{array}$$

(A76) $$\begin{array}{cc} a(\infty ) & =a_{0}\\ g(\infty ,\tau ) & =1\\ g_{0}\left(\infty \right)+e^{-\gamma \tau }G\left(\infty ,\tau \right) & =1\\ 1+e^{-\gamma \tau }G\left(\infty ,\tau \right) & =1\\ G(\eta ,\tau ) & \to 0\;\;\;\;\;\;\;\;\mathrm{as}\;\eta \to \infty . \end{array}$$

Then, τ is set to zero, and $F=F_{0}\left(\eta \right)$ and $G=G_{0}\left(\eta \right)$. Equations (A70) and (A71) become the following linear eigenvalue problem:

(A77) $$\begin{array}{cc} \left(1+\frac{1}{\beta }\right)F_{0}^{\prime \prime \prime }+f_{0}F_{0}^{\prime \prime }+f_{0}^{\prime \prime }F_{0}-\left(2f_{0}^{\prime }+M^{2}-\gamma \right)F_{0}^{\prime } & =0, \end{array}$$

(A78) $$\begin{array}{cc} \frac{1}{Sc}G_{0}^{\prime \prime }+g_{0}^{\prime }F_{0}+f_{0}G_{0}^{\prime }-\left(K-4Kg_{0}+3Kg_{0}^{2}-\gamma \right)G_{0} & =0, \end{array}$$

and boundary conditions (A72)–(A76) become:

$$\begin{array}{c} F_{0}\left(0\right)=0,\;\;\;\;\;\;F_{0}^{\prime }\left(0\right)=\upsilon \left(1+\frac{1}{\beta }\right)F_{0}^{\prime \prime }\left(0\right),\;\;\;\;\;\;G_{0}^{\prime }\left(0\right)=K_{s}G_{0}\left(0\right),\\ F_{0}^{\prime }\left(\eta \right)\to 0,\;\;\;\;\;\;G_{0}\left(\eta \right)\to 0\;\;\;\;\mathrm{as}\;\;\;\;\;\eta \to \infty . \end{array}$$

Then, $F_{0}^{\prime }\left(\eta \right)\to 0$ as η→∞ are chosen to relax. Thus, Equations (A77) and (A78) are solved along the new boundary conditions,

$$\begin{array}{c} F_{0}\left(0\right)=0,\;\;\;\;\;F_{0}^{\prime }\left(0\right)=\upsilon \left(1+\frac{1}{\beta }\right)F_{0}^{\prime \prime }\left(0\right),\;\;\;\;\;G_{0}^{\prime }\left(0\right)=K_{s}G_{0}\left(0\right),\;\;\;\;\;F_{0}^{\prime \prime }\left(0\right)=1,\\ G_{0}\left(\eta \right)\to 0\;\;\;\;\;\mathrm{as}\;\;\;\;\;\eta \to \infty . \end{array}$$

## References

[1] Mukhopadhyay S. (2013). Casson Fluid Flow and Heat Transfer over a Nonlinearly Stretching Surface. Chin. Phys. B.

[2] Pramanik S. (2014). Casson Fluid Flow and Heat Transfer Past an Exponentially Porous Stretching Surface in Presence of Thermal Radiation. Ain Shams Eng. J..

[3] Khan K.A., Butt A.R., Raza N. (2018). Effects of Heat and Mass Transfer on Unsteady Boundary Layer Flow of a Chemical Reacting Casson Fluid. Results Phys..

[4] Mahmoodi M., Esfe M.H., Akbari M., Karimipour A., Afrand M. (2015). Magneto-Natural Convection in Square Cavities with a Source-Sink Pair on Different Walls. Int. J. Appl. Electrom..

[5] Rashidi M.M., Abelman S., Freidooni Mehr N. (2013). Entropy Generation in Steady MHD Flow due to a Rotating Porous Disk in a Nanofluid. Int. J. Heat Mass Transf..

[6] Karimipour A., Esfe M.H., Safaei M.R., Semiromi D.T., Jafari S., Kazi S.N. (2014). Mixed Convection of Copper–Water Nanofluid in a Shallow Inclined Lid Driven Cavity using the Lattice Boltzmann Method. Phys. A Stat. Mech. Appl..

[7] Esfe M.H., Arani A.A.A., Karimiopour A., Esforjani S.S.M. (2014). Numerical Simulation of Natural Convection around an Obstacle Placed in an Enclosure Filled with Different Types of Nanofluids. Heat Transf. Res..

[8] Karimipour A., Nezhad A.H., D’Orazio A., Esfe M.H., Safaei M.R., Shirani E. (2015). Simulation of Copper–Water Nanofluid in a Microchannel in Slip Flow Regime using the Lattice Boltzmann Method. Eur. J. Mech. B/Fluids.

[9] Bahrami M., Akbari M., Karimipour A., Afrand M. (2016). An Experimental Study on Rheological Behavior of Hybrid Nanofluids Made of Iron and Copper Oxide in a Binary Mixture of Water and Ethylene Glycol: Non-Newtonian Behavior. Exp. Therm. Fluid Sci..

[10] Zadkhast M., Toghraie D., Karimipour A. (2017). Developing a New Correlation to Estimate the Thermal Conductivity of MWCNT-CuO/Water Hybrid Nanofluid via an Experimental Investigation. J. Therm. Anal. Calorim..

[11] Rashidi M.M., Kavyani N., Abelman S. (2014). Investigation of Entropy Generation in MHD and Slip Flow over a Rotating Porous Disk with Variable Properties. Int. J. Heat Mass Transf..

[12] Nadeem S., Haq R.U., Lee C. (2012). MHD Flow of a Casson Fluid over an Exponentially Shrinking Sheet. Sci. Iran..

[13] Raju C.S.K., Sandeep N., Sugunamma V., Babu M.J., Reddy J.V.R. (2016). Heat and Mass Transfer in Magnetohydrodynamic Casson Fluid over an Exponentially Permeable Stretching Surface. Eng. Sci. Technol. Int. J..

[14] Reddy P.B.A. (2016). Magnetohydrodynamic Flow of a Casson Fluid over an Exponentially Inclined Permeable Stretching Surface with Thermal Radiation and Chemical Reaction. Ain Shams Eng. J..

[15] Medikare M., Joga S., Chidem K.K. (2016). MHD Stagnation Point Flow of a Casson Fluid over a Nonlinearly Stretching Sheet with Viscous Dissipation. Am. J. Comput. Math..

[16] Qing J., Bhatti M.M., Abbas M.A., Rashidi M.M., Ali M.E.S. (2016). Entropy Generation on MHD Casson Nanofluid Flow over a Porous Stretching/Shrinking Surface. Entropy.

[17] Merkin J.H. (1996). A model for isothermal homogeneous-heterogeneous reactions in boundary-layer flow. Math. Comput. Model..

[18] Rana S., Mehmood R., Akbar N.S. (2016). Mixed Convective Oblique Flow of a Casson Fluid with Partial Slip, Internal Heating and Homogeneous–Heterogeneous Reactions. J. Mol. Liq..

[19] Raju C.S.K., Sandeep N., Saleem S. (2016). Effects of Induced Magnetic Field and Homogeneous–Heterogeneous Reactions on Stagnation Flow of a Casson Fluid. Eng. Sci. Technol. Int. J..

[20] Sheikh M., Abbas Z. (2017). Homogeneous–Heterogeneous Reactions in Stagnation Point Flow of Casson Fluid due to a Stretching/Shrinking Sheet with Uniform Suction and Slip Effects. Ain Shams Eng. J..

[21] Khan M.I., Waqas M., Hayat T., Alsaedi A. (2017). A Comparative Study of Casson Fluid with Homogeneous-Heterogeneous Reactions. J. Colloid Interface Sci..

[22] Khan M.I., Hayat T., Khan M.I., Alsaedi A. (2017). A Modified Homogeneous-Heterogeneous Reactions for MHD Stagnation Flow with Viscous Dissipation and Joule Heating. Int. J. Heat Mass Transf..

[23] Merkin J.H. (1986). On Dual Solutions Occurring in Mixed Convection in a Porous Medium. J. Eng. Math..

[24] Weidman P.D., Kubitschek D.G., Davis A.M.J. (2006). The Effect of Transpiration on Self-Similar Boundary Layer Flow over Moving Surfaces. Int. J. Eng. Sci..

[25] Mahapatra T.R., Nandy S.K. (2011). Stability Analysis of Dual Solutions in Stagnation-Point Flow and Heat Transfer over a Power-Law Shrinking Surface. Int. J. Nonlinear Sci..

[26] Roşca A.V., Pop I. (2013). Flow and Heat Transfer over a Vertical Permeable Stretching/Shrinking Sheet with a Second Order Slip. Int. J. Heat Mass Transf..

[27] Awaludin I.S., Weidman P.D., Ishak A. (2016). Stability Analysis of Stagnation-Point Flow over a Stretching/Shrinking Sheet. AIP Adv..

[28] Chaudhary M.A., Merkin J.H. (1995). A Simple Isothermal Model for Homogeneous-Heterogeneous Reactions in Boundary-Layer Flow. I Equal Diffusivities. Fluid Dyn. Res..

[29] Harris S.D., Ingham D.B., Pop I. (2009). Mixed Convection Boundary-Layer Flow Near the Stagnation Point on a Vertical Surface in a Porous Medium: Brinkman Model with Slip. Transp. Porous Media.

[30] Bilal M., Hussain S., Sagheer M. (2017). Boundary Layer Flow of Magneto-Micropolar Nanofluid Flow with Hall and Ion-Slip Effects using Variable Thermal Diffusivity. Bull. Pol. Acad. Sci. Tech. Sci..

[31] Kameswaran P.K., Shaw S., Sibanda P. (2014). Dual Solutions of Casson Fluid Flow over a Stretching or Shrinking Sheet. Sadhana.

[32] Bhattacharyya K. (2011). Dual Solutions in Boundary Layer Stagnation-Point Flow and Mass Transfer with Chemical Reaction past a Stretching/Shrinking Sheet. Int. Commun. Heat Mass.

[33] Miklavčič M., Wang C. (2006). Viscous Flow due to a Shrinking Sheet. Q. Appl. Math..

